# Water-mediated ribonucleotide–amino acid pairs and higher-order structures at the RNA–protein interface: analysis of the crystal structure database and a topological classification

**DOI:** 10.1093/nargab/lqae161

**Published:** 2024-12-11

**Authors:** Raman Jangra, John F Trant, Purshotam Sharma

**Affiliations:** Computational Biochemistry Laboratory, Department of Chemistry and Centre for Advanced Studies in Chemistry, Panjab University, Sector 14, Chandigarh 160014, India; Department of Chemistry and Biochemistry, University of Windsor, 401 Sunset Ave. Windsor, ON, N9B 3P4, Canada; We-Spark Health Institute, University of Windsor, 401 Sunset Ave. Windsor ON, N9B 3P4, Canada; Binary Star Research Services, University of Windsor, LaSalle, ON, N9J 3X8, Canada; Computational Biochemistry Laboratory, Department of Chemistry and Centre for Advanced Studies in Chemistry, Panjab University, Sector 14, Chandigarh 160014, India; Department of Chemistry and Biochemistry, University of Windsor, 401 Sunset Ave. Windsor, ON, N9B 3P4, Canada

## Abstract

Water is essential for the formation, stability and function of RNA–protein complexes. To delineate the structural role of water molecules in shaping the interactions between RNA and proteins, we comprehensively analyzed a dataset of 329 crystal structures of these complexes to identify water-mediated hydrogen-bonded contacts at RNA–protein interface. Our survey identified a total of 4963 water bridges. We then employed a graph theory-based approach to present a robust classification scheme, encompassing triplets, quartets and quintet bridging topologies, each further delineated into sub-topologies. The frequency of water bridges within each topology decreases with the increasing degree of water node, with simple triplet water bridges outnumbering the higher-order topologies. Overall, this analysis demonstrates the variety of water-mediated interactions and highlights the importance of water as not only the medium but also the organizing principle underlying biomolecular interactions. Further, our study emphasizes the functional significance of water-mediated interactions in RNA–protein complexes, and paving the way for exploring how these interactions operate in complex biological environments. Altogether, this understanding not only enhances insights into biomolecular dynamics but also informs the rational design of RNA–protein complexes, providing a framework for potential applications in biotechnology and therapeutics. All the scripts, and data are available at *https://github.com/PSCPU/waterbridges*.

## Graphical Abstract

**Graphical Abstract:**
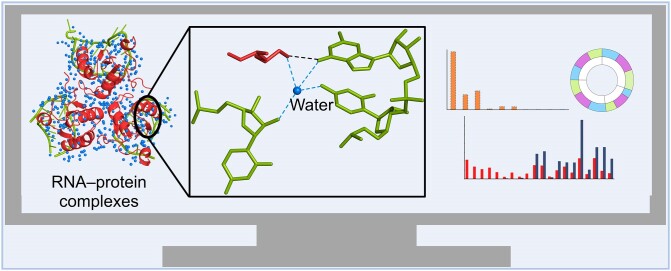

## Introduction

RNA–protein recognition potentiates life: fundamental cellular process like translation, transcription, transfer RNA (tRNA) loading, mRNA splicing and post-transcriptional regulation of gene expression all rely on this phenomenon (1–3). The RNA-first hypothesis suggests that these are the evolutionarily earliest interactions between two classes of biomolecules and this supramolecular biochemistry underlies the rest of biology (4,5). Recognition of RNA by proteins is facilitated by plethora of noncovalent interactions, the reversible nature of which allows them to exist and function without affecting molecular integrity. Such interactions include (direct and water-mediated) hydrogen bonding, van der Waals interactions, hydrophobic interactions, π-interactions, ionic interactions and stacking interactions (6). Hydrogen-bonds are the most important, being very strong, directional and requiring specific conditions for formation (i.e. the presence of suitable acceptor and donor atoms in the appropriate geometrical orientation) (7,8). This ensures that specific conformations are adopted by these systems.

Direct RNA–protein hydrogen bonds serve diverse important functions, as varied as the encapsidation of the Vesicular Stomatitis Virus genomic RNA by its nucleocapsid protein and the production of templates that support RNA synthesis (9), the specific binding of the HIV-1 Tat protein and its TAR RNA through arginine fork during transcription (10), and the recognition of the 5S rRNA by the Cys2-His2 zinc fingers of transcription factor IIIA (TFIIIA) (11,12). Consequently, it is unsurprising that there are numerous theoretical studies based on the identification, characterization and contextual analysis of direct hydrogen bonds in the crystal structures of RNA–protein complexes available (13–20). Even though they are all of the same vintage (2001–2008), they have many mutual discrepancies due to differential identification criteria adopted, and the variable number and cut-off resolution of the crystal structures investigated. This explosion of studies was followed by a hiatus, felt more broadly across structural molecular biology as the simple collection of data, such as the human genome project, failed to provide immediate payoff (21). This was always to be expected: a careful analysis of the big data coupled with a discovery of use cases is necessary to realize the promise of structural biology; consequently, this question was recently revisited to address the discrepancies of these previous studies and establish a reliable and up-to-date anatomy of direct RNA–protein hydrogen bonds (22). This emphasized the dominant role of the phosphate moiety of ribonucleotides in hydrogen bonding, and further revealed that hydrogen bonds between Arg and rA are the most frequent specific interactions between amino acids and RNA (22). Unfortunately, studying direct interactions alone might overlook some of the most important organizing intramolecular interactions.

Despite significant progress towards understanding the role of direct hydrogen bonds, the occurrence and structural role of water-mediated hydrogen bonds in the context of RNA–protein recognition has been largely ignored. More broadly, the role of water in biomolecular interactions is often de-emphasized, potentially because with the emergence of *in silico* tools, the comparative difficulty of considering specific and non-specific, and highly dynamic water interactions is extremely computationally expensive: it is easier and has been more efficient to simply de-emphasize the role of water so as to justify not incorporating it into models (23,24). Furthermore, even modern tools, more than capable of considering explicit water, are generally built on top of legacy algorithms that didn’t for feasibility reasons.

**Figure 1.**
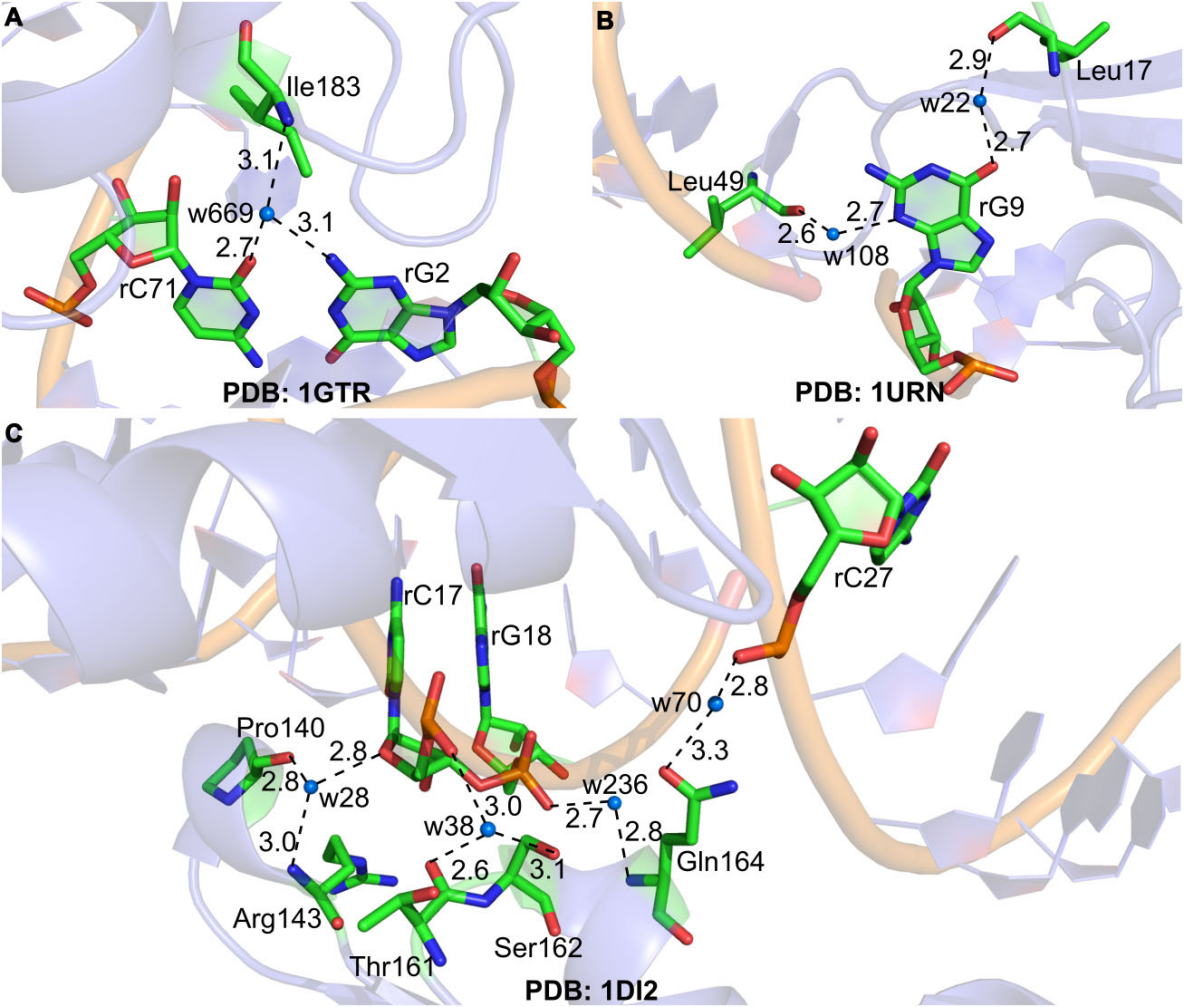
Importance of water molecules in the recognition of RNA by proteins. (**A**) Water bridge between Ile183, rG2 and rC71 facilitates tRNA^Gln^ recognition by *Escherichia coli* glutaminyl-tRNA synthetase (27,28). (**B**) Water bridges between Leu17, Leu49 and rG9 are crucial for binding of U1A protein to its pre-mRNA (29,30). (**C**) Multiple water bridges promote *Xenopus laevis* RNA-binding protein A (Xlrbpa-2) to dsRNA (31). In all structures, water molecules (w) are represented as marine blue spheres. Donor–acceptor (D–A) distances (Å) are provided for each water-mediated interaction.

Although water exhibits a three-dimensional tetrahedral network-like structure and can thereby help mediate specific molecular recognition (25), most water-mediated hydrogen bonds in DNA–protein complexes were often previously regarded as highly degenerate, non-specific and mere space-fillers (26). However, with the surge in the availability of high-resolution crystal structures of RNA–protein complexes over the past two decades resulting from significant improvements in X-ray techniques for atomic-level structure elucidation, it is clear this is not the case. In fact, crystallography has highlighted functionally important examples of water-mediation in RNA–protein complexes. For example, water bridging between Ile183, rG2 and rC71 forms a recognition surface for rG2·rC71 and rG3·rC70 base pairs during tRNA^Gln^ recognition by *Escherichia coli* glutaminyl-tRNA synthetase (GlnRS, PDB: *1GSG*, Figure 1A) (27,28). Similarly, a water molecule facilitates the binding of U1A protein to its pre-mRNA, by bridging rG9, Leu17 and Leu49 (PDB: *1URN*, Figure 1B) (29,30); this binding is pivotal for the regulatory function of the U1A protein in the production of its own mRNA (29,30). Water molecules, likewise, help in the specific binding of various proteins to RNA, like the binding of *Xenopus laevis*’ RNA-binding protein A (Xlrbpa-2) to dsRNA (PDB: *1DI2*, Figure 1C) (31), human signal recognition particle 19’s (SRP19) to the SRP RNA sequence (PDB: *1JID*, Supplementary Figure S1A) (32), *Methanococcus jannaschii* SRP54’s to SRP RNA (PDB: *2V3C*, Supplementary Figure S1B) (33), cytoplasmic protein ZC3H12B to RNA (PDB: *6SJD*, Supplementary Figure S1C) (34) and *Mycobacterium tuberculosis’* Phe-tRNA synthetase to the tRNA^Phe^ (PDB: *7KA0*, Supplementary Figure S1D) (35). This is part of the general re-evaluation of water’s structure; what was once thought to be a random dynamic process is now understood to be a series of rapidly interconverting and dynamic clusters (36–38); it is unsurprising that water is also central to the organization of biomolecules. Water forms directional interactions; even those that are not conserved in the traditional crystallographic sense are involved in predictable dynamic interactions.

Owing to their inherent diversity, water-mediated RNA–protein interactions can be classified based on their structural characteristics. Although a detailed, unambiguous classification of water-mediated interactions in RNA–protein complexes is not available, the role of water has, nevertheless, been appreciated in the context of ligand–protein (39) and DNA–protein complexation (40,41). Nevertheless, in context of RNA–protein complexes, Li *et al.* categorized water-mediated contacts in terms of the number of waters participating in a bridge, considering that many such contacts are wider than the diameter of a single water molecule (42). However, this classification scheme is limited since it does not consider multivalent water bridges. Specifically, despite their relative abundance, divalent water molecules simply bridging a single ribonucleotide–amino acid pair may not be the most important mechanism for molecular recognition specificity; higher-ordered structures involving a network of hydrogen bonds connected through multiple ribonucleotides and amino acid residues and involving highly confined water are both more likely to be conserved and potentially more critical in RNA–protein recognition.

A few previous studies tried to untangle and describe the interactions between molecular statistics, structural characteristics and the functional role of water-mediated bonds in RNA–protein crystal structures, although the analysis has always been hampered by the limited availability of crystal structures. In 1998, Nadassy *et al.* analyzed 75 protein-nucleic acid structures and established the abundance of polar water-mediated interactions and affirmed their critical role in facilitating close packing (43). Subsequently, in 2001, Treger *et al.* visually analyzed 45 crystal structures, of which 17 complexes contained 309 water-mediated hydrogen bonds (16). In 2003, Jeong *et al.* analyzed 51 RNA–protein complexes and identified 1276 water-mediated hydrogen bonds in comparison to 1568 direct hydrogen bonds (17). In addition, in 2011 Kondo *et al.* visually identified 22 water-mediated pseudo pairs constituted by Asn, Gln, Arg, Asp and Glu amino acids and ribonucleotides in 442 RNA–protein complexes (44). We note of course that any study (including this one) is simply a snapshot in time, and all such studies need to be revisited on a regular basis.

Emphasizing this point, there has been an exponential increase in the rate of the deposition of new RNA–protein complexes in the (PDB), almost quadrupling the available data in the past decade (45). This expansion has enhanced the fair representation of various RNA and protein types. However, despite the enhanced count and diversity of RNA–protein complexes in the now-available larger dataset, the most recent PDB survey for water bridges was performed in 2014 by Barik *et al.*, where a total of 878 bridging waters were identified in 89 RNA–protein complexes (46). It is unclear if the distribution of the data has changed: the complexes that were studied in the 1990s and 2000s might differ from those now being deposited, or we might simply be increasing the number of structures of the same complexes in a consistent ratio. This bioinformatics analysis is a topic for a separate study, but quadrupling the number of structures, and increasing the resolution of the average structure due to improvements in both crystallization methods and instrumentation, means that the statistics can be expected to have changed. We also need to generate a classification system that both encapsulates the simple divalent bridges captured in previous studies, but also adds the capacity to highlight the up to pentavalent water molecules that can be found in some structures.

In the present work, we carry out a detailed analysis of water-mediated hydrogen bonding contacts at the RNA–protein interface. We first compare the distribution of direct and water-mediated hydrogen bonding interactions in a carefully crafted dataset of up-to-date non-redundant high resolution X-ray structures of the RNA–protein complexes. We then propose a graph theory-based hierarchical classification of these contacts. In analogy with the existing classification schemes for base paring (47) and T-shaped interactions in RNA (48), the contacts involving the nucleobase moiety of ribonucleotide are then subclassified based on the nucleobase edge, i.e. Watson-Crick (WC) edge, Hoogsteen (HG) edge or Sugar (SG) edge and the portion of amino acid (main chain [m] or side chain [s]) that interacts with the bridging water (Supplementary Figures S2 and S3). Overall, our analysis is expected to contribute to a better understanding of the physicochemical forces involved in RNA–protein recognition and the consideration of these effects in the emerging field of structural synthetic biology.

## Material and methods

### Dataset preparation

All 3D structures of RNA–protein complexes elucidated using X-ray diffraction with better than 2.5 Å refinement resolution released on or before 15 March 2022 and containing at least one water molecule, were extracted from the Protein Data Bank (PDB), using the ‘Advanced Search’ query builder available on the PDB website (https://www.rcsb.org/) (49,50). For this, the ‘Polymer Entity Type’ query was fixed to ‘Protein’ and ‘RNA,’ and structures containing DNA and nucleic-acid hybrids were excluded using further subqueries. This resulted in a dataset containing 705 crystal structures. To eliminate redundancy, RNA–protein complexes having >30% sequence identity were removed from our dataset using the CDHIT suite (22,44,45). This process provided a nonredundant dataset of 329 crystal structures (Supplementary Table S1).

### Identification and classification of water-mediated motifs

**Figure 2.**
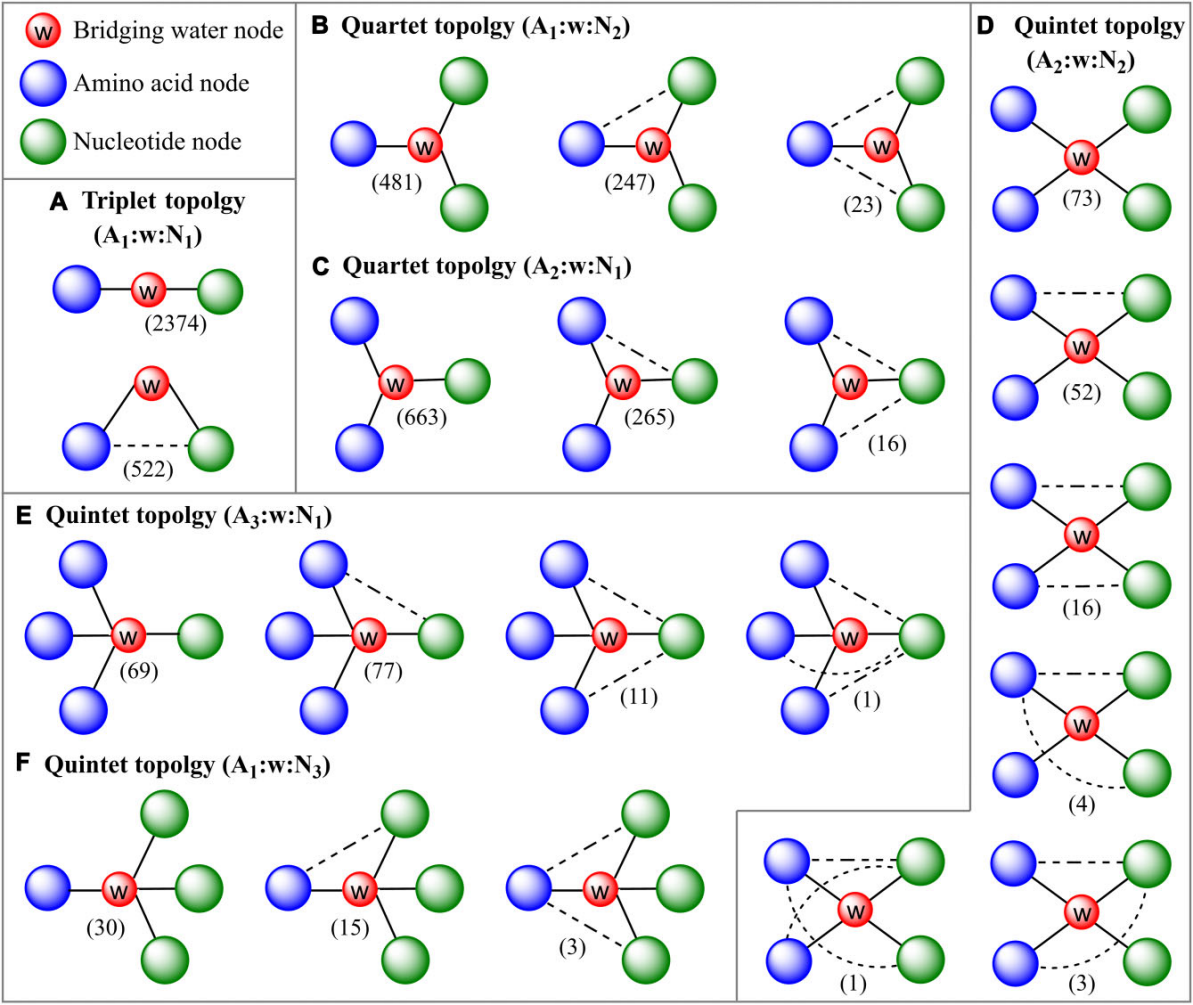
Identified topologies of water-mediated motifs. Solid lines represent hydrogen bonds with water molecule (red nodes), and dotted lines represent direct hydrogen bonds between amino acids (blue nodes) and nucleotide (green nodes). The frequency of each topology is indicated in parentheses, and additional PDB-specific frequencies are available in the Supplementary Excel file.

HBPLUS was used for the identification of hydrogen bonds (51), since it uses a robust algorithm for estimating the positions and geometries of all hydrogens, assisting with identification of hydrogen bonds. Apart from its robust and customizable algorithm, HBPLUS has been extensively used by many researchers for studying hydrogen bonding in RNA–protein complexes (6,15,17,22,46). In synchrony with previous studies (16,46), the donor heteroatom–acceptor heteroatom (D–A) distance, H–A distance and D–H–A angle cut offs were set to 3.35 Å, 2.7 Å and 90°, respectively, to eliminate serendipitous long-distance interactions. The execution of HBPLUS was automated using python scripts to process multiple PDB files. An additional python script was developed to partition the water–ribonucleotide hydrogen bonds and water–amino acid hydrogen bonds into two categories. Then any water molecule that appeared in both lists, implying it forms at least one bond with an amino acid and at least one bond with a ribonucleotide was clubbed. A third python script was written to automate the labeling of the graph theory-based topology of water-mediated motifs by counting the unique amino acid and ribonucleotide residues involved in their formation (*vide infra*, Figure 2). These scripts are all available on GitHub (*https://github.com/PSCPU/waterbridges*) and easily installable via pip (https://pypi.org/project/waterbridge/).

### Sub-classification of water-mediated topologies

The topological classification incorporates the identity of the ribonucleotide (nucleobase [Nb], ribose [Rb] or phosphate [Ph]) and amino acid (including whether it is a main chain [m] or side chain [s]) portion that interact with the bridging water (Supplementary Figures S2 and S3). Interactions involving nucleobases were subclassified based on the interacting nucleobase edges (Watson–Crick [WC], Hoogsteen [HG] and Sugar [SG], Supplementary Figure S2). Some atoms, associated with more than one edge, induce ambiguity in the choice of interacting edge, especially in those case where the nucleobase forms only one hydrogen bond with bridging water (Supplementary Table S2). To address this, we implemented a lucid algorithm in our python code based on the assertion that a bridging water molecule can form hydrogen bonds with at most two atoms of a single nucleobase moiety due to geometrical constraints (*vide infra*).

Initially, we created a sorted list (*int_atom*) of atoms of a nucleobase interacting with the bridging water. Two cases arise here. In the first case, two atoms interact with water. In this case, the list (*int_atom*) will contain two atoms. The possible edgewise combinations of two atoms are [*N1*, *N6*], [*N1*, *N2*], [*N1*, *O6*], [*N3*, *N4*], [*N3*, *O2*] and [*N3*, *O4*] for the WC edge (*wc_list*); [*N6*, *N7*] and [*N7*, *O6*]) for the HG edge (*hg_list*); and [*N3*, *N9*], [*N3*, *O2′*], [*N1*, *O2*], [*N2*, *N3*] and [*O2*, *O2′*] for the SG edge (*sg_list*, Supplementary Figure S2). Here, atom *O2′* is included in the SG edge by default (52). However, if *O2′* interacts in combination with *O3′*, then the interaction will be considered as ribose-sugar mediated, rather than nucleobase mediated. We then visually inspected the association of the initially extracted list (*int_atom*) with its corresponding edge to determine which nucleobase edge is formally involved in the water bridge formation.

In the second case, only one nucleobase atom interacts with the bridging water. This in turn results in two subcases. In the first subclass, the ribonucleotide edge can be unambiguously assigned. This subclass includes atoms that unambiguously belong to a specific nucleobase edge. For example, if any water molecule interacts with *N1* of purine, the interacting edge can be unambiguously assigned as WC. Similarly, the *N7* atom unambiguously belongs to the HG edge (Supplementary Figure S2). Additionally, when atom *O2′* interacts solely with a water molecule (i.e. without *O3′* involvement), it is considered to be associated with the SG edge. The second subcase involves atoms for which the edge cannot be unambiguously assigned (marked with # in Supplementary Figure S2 and Supplementary Table S2). To overcome this ambiguity, we calculated the Euclidean distance of oxygen atom of bridging water molecule from two atoms immediate adjacent to the atom under consideration:


\begin{eqnarray*}d\left( {{\mathrm{a}},{\mathrm{\;O}}} \right) = {\mathrm{\;}}\sqrt {{{\left( {{{\mathrm{x}}_{\mathrm{a}}} - {{\mathrm{x}}_{\mathrm{O}}}} \right)}^2}{\mathrm{\;}} + {\mathrm{\;}}{{\left( {{{\mathrm{y}}_{\mathrm{a}}} - {{\mathrm{y}}_{\mathrm{O}}}} \right)}^2} + {{\left( {{{\mathrm{z}}_{\mathrm{a}}} - {{\mathrm{z}}_{\mathrm{O}}}} \right)}^2}} \end{eqnarray*}

Here, x_a_, y_a_, z_a_, x_O_, y_O_ and z_O_ are the extracted cartesian coordinates from the PDB file. For example, for the categorization of water bridge involving only *N6* of rA, we calculated the distance of oxygen atom of water molecule from two adjacent atoms of *N6*, i.e*. C5* and *N1* and if d(*N1*,O) < d(*C5*,O); then water bridge belongs to the Watson-Crick edge, else the Hoogsteen edge is assigned to the bridge. A similar approach is used for all atoms marked by # in Supplementary Figure S2.

## Results

### Non-degenerate comprehensive dataset reveals the frequency of water bridges between various RNA and protein types

The analyzed dataset of 329 high resolution X-ray structures of RNA–protein complexes, involves 20 different RNA types and 23 protein types (Supplementary Tables S3 and S4). The numerically predominant RNA types include single-stranded RNA (ssRNA, 39.5%), tRNA (10.6%) and double-stranded RNA (dsRNA,10.0%) while nucleic acid binding protein (35.0%), hydrolases (22.2%) and transferases (9.7%) constitute the major protein types (Supplementary Tables S3 and S4). The dataset is inherently biased by the terms of availability of specific RNA and protein types.

We identified a total of 106 887 crystallographic water molecules in the 329 crystal structures (Supplementary Table S1). This equates to an average of 325 water molecules per crystal structure, but the variance is large ranging from 6 to 7893 waters per crystal strucutre (Supplementary Table S1). Of these, only 4693 water molecules (0.04% of the total), spanning 305 crystal structures, participate in an inter-biomolecule water bridge with at least one hydrogen bond with RNA and at least one hydrogen bond with a protein (Supplementary Table S1). Approximately 97.6% of such waters form up to four hydrogen bonds, whereas the remaining 2.4% form more than four hydrogen bonds. This is higher than the tetrahedral limit and may possibly occur due to ambiguity in the location of atomic positions within crystal structures (Supplementary Table S5), although non-classical bifurcated interactions are possible (53–55). These bridging water molecules form 6746 hydrogen bonds with 5338 amino acid residues, and 6686 hydrogen bonds with 3554 ribonucleotides (Supplementary Tables S6 and S7). On average each interacting amino acid forms approximately 1.2 hydrogen bonds with each bridging water (Supplementary Table S6), whereas each interacting ribonucleotide forms two hydrogen bonds with a bridging water, suggesting greater affinity of water for the ribonucleotides compared to amino acids (Supplementary Table S7).

### Purines and acidic amino acids dominate in water bridges

**Figure 3.**
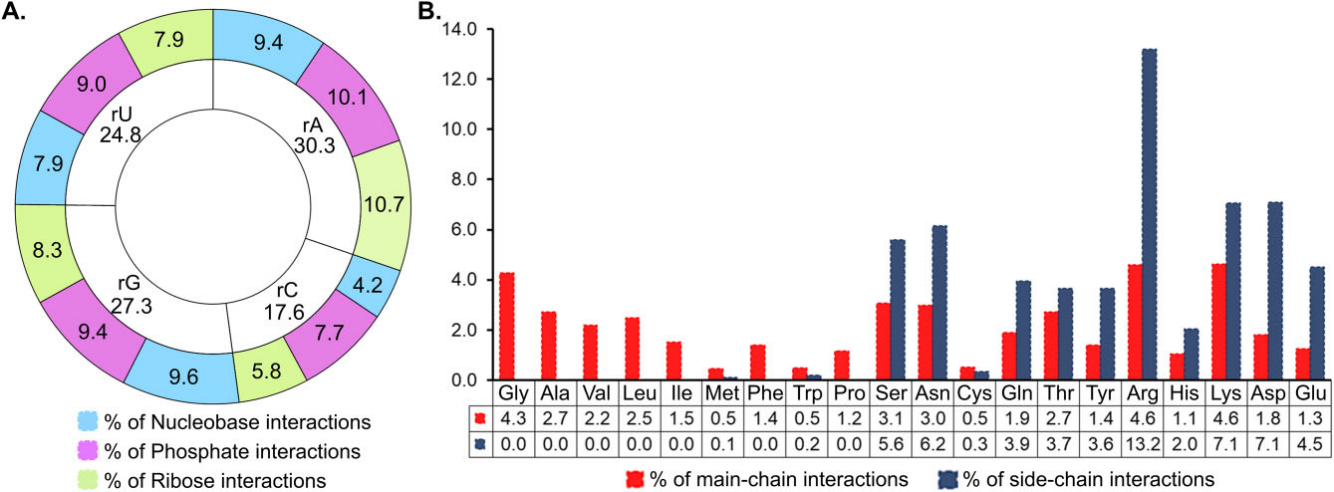
Frequency distribution of the hydrogen bonds formed by (**A**) ribonucleotides and (**B**) amino acids with bridging waters as a function of their respective interacting moieties.

**Figure 4.**
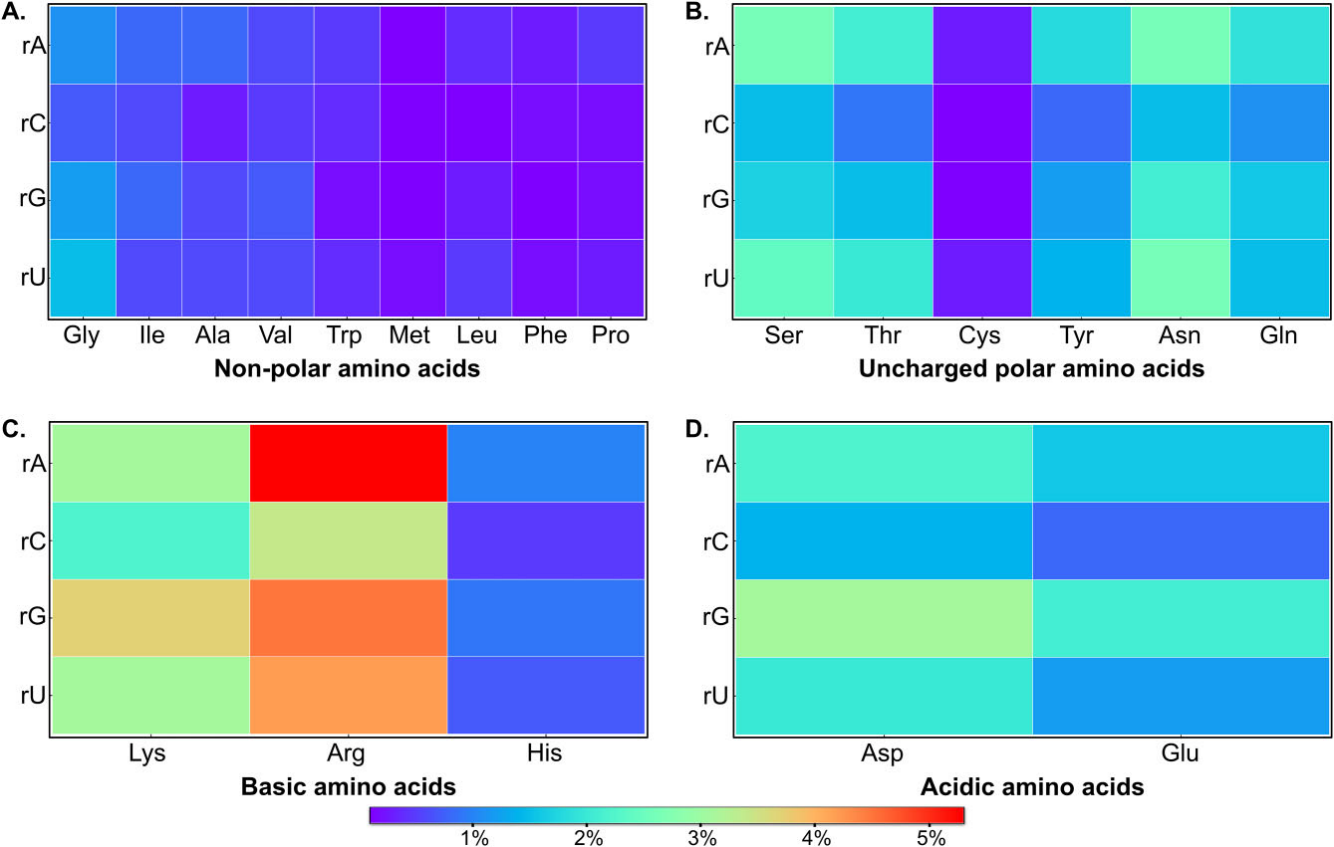
Visualizations of the frequency of pairs of amino acids and ribonucleotides involved in water bridge formation. The color scale at the bottom expresses the percentage contribution of each pair.

Inspecting the distribution of the bridging hydrogen bonds as a function of ribonucleotide identity reveals that these interactions most commonly involve rA (30.3%), followed by rG (27.3%), rU (24.8) and rC (17.6%), which suggests a significant preference for purines (57.6%) over pyrimidines (42.4%, Figures 3A and 4; Supplementary Table S7). However, when comparing the different moieties of the nucleotide, we see an even split between phosphate (36.2%), ribose (32.7%) and nucleobase (31.1%) moieties (Figure 3A and Supplementary Table S7). In terms of hydrogen bonds involving the nucleobase moiety, G (30.8%) and A (30.3%) contribute the most, followed by U (25.5%) and C (13.4%, Figure 3A and Supplementary Table S7). Such hydrogen bonds are predominantly formed by endocyclic nitrogen atoms (43.0%), followed by carbonyl oxygens (38.4%) and exocyclic amino nitrogen (18.8%, Supplementary Table S8). In addition, the 2′-OH of the ribose moiety contributes 64.9% to sugar-specific and 37.7% to the total water-mediated contacts (Supplementary Table S8).

In terms of protein constituents, 17.8% of the interactions are formed by Arg, followed by Lys (11.7%) and Asn (9.1%, Figures 3B and 4; Supplementary Table S6). Overall, the polar amino acids account for 83.1% of all interactions, with charged polar side chains contributing 12.7% more than the neutral sidechains (Figure 3B and Supplementary Table S6). Furthermore, side chain atoms have slightly greater contribution to these hydrogen bonds (57.5%), compared to the main chain atoms (Supplementary Table S6), although main-chain carbonyl oxygen (26.8%) and amino nitrogen (15.5%) participate more frequently in water bridge formation than any other amino-acid atom (Supplementary Table S9). To be fair, these are present in every single residue. A very small fraction of bridging water and amino acid residues involve the sulfur atoms of Cys and Met (0.4%, Supplementary Table S9).

Furthermore, in terms of the frequency of ribonucleotide-amino acid pairs involved in water bridge formation, the rU-Gly pair (1.5%) is the most frequent among non-polar amino acids, while rA-Ser (2.6%) and rU-Asn (2.6%) dominate among uncharged polar amino acids (Figure 4). The rA-Arg pair (5.3%) is the most common among basic amino acids, and the rG-Asp pair (3.1%) is the most frequent among acidic amino acids (Figure 4).

### Water bridges can be categorized as multiplets based on topology

**Figure 5.**
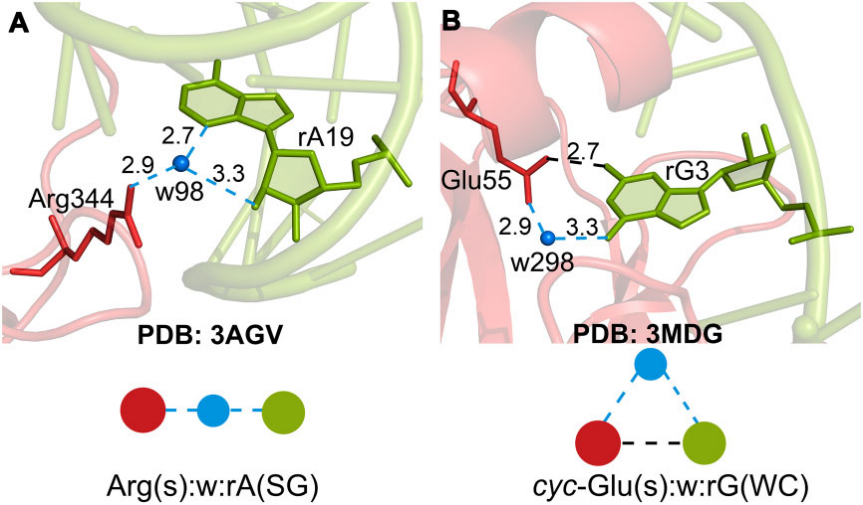
Examples of triplet water bridges. (**A**) An A_1_:w:N_1_ water bridge in the crystal structure of the Fc fragment of human IgG1 (hFc1) complexed with an anti-Fc RNA aptamer. (**B**) An *cyc-*A_1_:w:N_1_ water bridge in Human cleavage factor, *Im* in complex with RNA. Water molecules are represented in blue. Blue vertices/dashed lines indicate water bridges, while black vertices represent direct hydrogen bonds between amino acids (in red) and ribonucleotides (in green).

In many cases, complex networks of water-mediated interactions exist in the crystal structures of RNA–protein complexes. As a result, a classification scheme is necessary to understand the explicit role of water in facilitating these nucleic acid–protein recognition events. We propose that these can be classified using graph theory (56–58). Within this framework, a water bridge can be described as a multiplet: each interacting amino-acid residue, ribonucleotide and water molecule is represented by a node, a hydrogen bond between two entities is represented by a vertex, and the number of vertices attached to a single node describe the degree of the node (Figures 2 and 5). The order of the multiplet is determined by the total number of entities (including the central water) that participate in the formation of the water bridge. Accordingly, water bridges may be contemplated as multiplets of various order, namely triplet, quartet, quintet, etc., and their topologies are defined by the number of interacting amino acids and ribonucleotides (Figure 2).

To illustrate this scheme, let us consider the simplest water bridge consisting of a single ribonucleotide and a single amino acid, where the ribonucleotide and amino acid interact only through water mediation. This bridge belongs to the triplet topology, as it encompasses three nodes representing one ribonucleotide, one amino acid, and the bridging water (Figures 2A and 5A). Here, the water node will have a degree of two, while the amino acid and nucleotide nodes will have a degree of one. Thus, each multiplet has an order one greater than the degree of the respective water node. The triplet can be denoted as A_1_:w:N_1_, where the numbers in subscript represent the number of amino acids (A) or ribonucleotides (N) involved in water bridge formation, and ‘w’ represents water. For example, a water molecule of degree two can form a triplet water bridge by interacting with rA and Arg (Figure 5A). Indeed, this is the dominant observed topology in water bridges and spans 58.3% of the total bridges (Supplementary Table S10).

**Figure 6.**
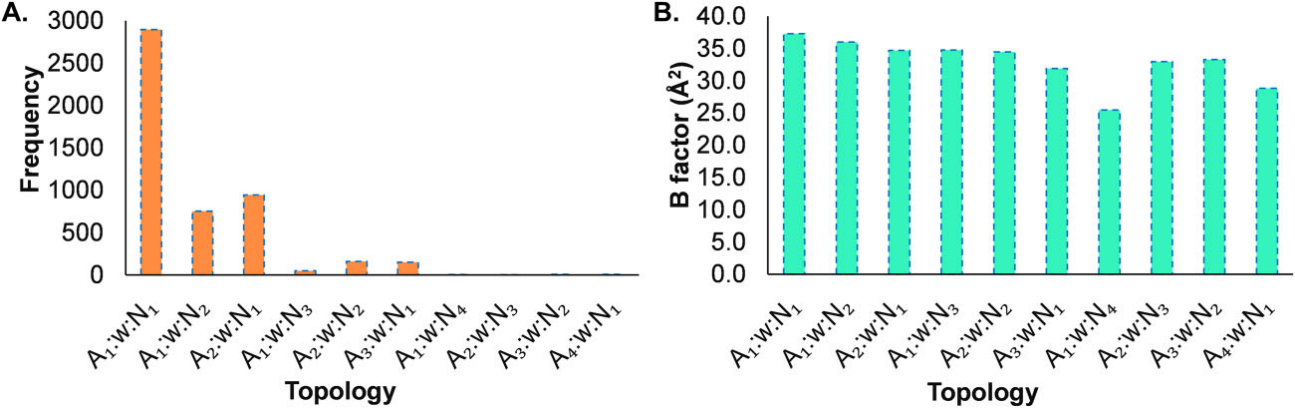
(**A**) Frequencies of the different water bridge topologies. (**B**) Relationship between the average B-factor and water bridge topology.

For higher order multiplets (i.e. with order > 3), many sub-topologies are possible within each topology. Specifically, a quartet topology can have two sub-topologies, since the water node of degree three can interact with either one amino acid and two ribonucleotides, or two amino acids and one ribonucleotide (Figure 2B and C). These sub-topologies can be represented as A_1_:w:N_2_ or A_2_:w:N_1_ respectively (Figure 2B and C; Supplementary Figures S4 and S5). The occurrence of these topologies (34.2%) is, however, significantly lower than A_1_:w:N_1_ (Supplementary Table S10). More importantly, this framework can be readily extended to classify other higher-ordered water bridges (Supplementary Figures S6–S9). However, topologies only up to a quintet are observed in our dataset of RNA–protein crystal structures (Supplementary Table S10); the frequency of a topology in the dataset is roughly inversely proportional to its order (Figure 6A).

### The topologies can be either cyclic or acyclic

The topological classification can be further extended by including the degree of the amino acid and ribonucleotide nodes. This results in acyclic and cyclic topologies (Figure 5). In acyclic topologies, all participating amino acid and ribonucleotide nodes are of degree 1, i.e. there is no direct interaction between the amino acids and ribonucleotides (Figure 5A). However, in the case of a cyclic topology, at least one direct hydrogen bond exists between an amino acid and a ribonucleotide involved in the water bridge (Figure 5B). In these examples, the degree of at least one amino acid and ribonucleotide pair will be greater than one. For example, the simplest cyclic topology (*cyc*) belongs to triplet water bridge (A_1_:w:N_1_), and is represented as *cyc*-A_1_:w:N_1_. This has the highest occurrence among all cyclic topologies (41.1%, Supplementary Table S10). This is followed by *cyc*-A_2_:w:N_1_ and *cyc*-A_1_:w:N_2_ topologies with almost equal frequency (22.1% and 21.3% respectively, Supplementary Table S10). A similar trend is also observed for the acyclic topologies, where triplet water bridges are most common (64.3%), followed by quartet water bridges (A_2_:w:N_1_ (18.0%) and A_1_:w:N_2_ (13.0%), Supplementary Table S10). Due to the involvement of direct hydrogen bonds providing a separate anchoring mechanism leading to multivalent binding, cyclic topologies are expected to be more stable than acyclic topologies, even though almost all acyclic topologies are more common than their cyclic counterparts (except in the case of quintet water bridges where the cyclic systems are more common, Supplementary Table S10). Overall, 25.6% of all water bridges in this sample are cyclic (Supplementary Table S10).

### Acyclic triplet multiplets dominate the dataset compared to higher-ordered water bridges but are also structurally different from the more complex systems

Although the triplet topologies are more common than the higher-ordered water bridges, the overall composition in terms of ribonucleotide and amino acid components largely remains similar (Supplementary Tables S11 and S12). However, the participation of the nucleobase moiety in water bridges decreases in higher-ordered bridges (29.0%) compared to in triplets (33.4%); instead, we observe an increase in ribose interactions (Supplementary Table S11). Furthermore, although the contribution of nucleobase A is greatest in triplet bridges (33.0%), G dominates in higher-ordered bridges (32.6%). The prevalence of cyclic topologies is notably greater in higher-ordered water bridges than in triplet systems (18.0%, Supplementary Table S10). Geometrically, there are a greater number of potential interactions in these larger systems, and less steric strain, facilitating ring-formation. We do not observe any real change in the nature of the amino acid moiety, with the backbone amide bonds accounting for the same percentage of interactions for both triplet and higher-order structures (42.9% and 42.2% respectively, Supplementary Table S12).

The average B-factor value of all bridging water topologies (36.3 Å^2^) is significantly lower than the overall average of all water molecules in the crystal structures (41.4 Å^2^, Supplementary Table S13). More importantly, water molecules involved in higher-ordered topologies possess a smaller average B factor (34.9 Å^2^) than those involved in triplet water bridges (37.3 Å^2^). This suggests that the water in higher-ordered topologies is more confined and may be functionally important (Figure 6B and Supplementary Table S13). Indeed, this is exemplified by a quartet water bridge between the human SRP19 protein and SRP RNA (PDB: *1JID*). Coupled with a separate triplet water bridge, this bridge is pivotal in facilitating the binding of the SRP19 protein to SRP RNA, a crucial step in SRP assembly in both *archaea* and *eukarya* (Supplementary Figure S1A) (32).

### Most triplet water bridges involve the nucleobase and can be classified by the edge involved

More than 50% of the 2896 triplet motifs use only the nucleobase moieties in bridge formation (1279 A_1_:w:N_1_ and 301 *cyc*-A_1_:w:N_1_ topologies, Tables 1 and 2; Supplementary Tables S14, S15). Considering the high frequency of nucleobase-based triplets, we developed a classification and nomenclature scheme specific to those that interact with a nucleobase (i.e. A_1_:w:N_1_ and *cyc*-A_1_:w:N_1_ topologies), although the concept can be easily extended to higher-ordered bridges.

**Table 1.** Frequency distribution of nucleobase-mediated acyclic triplet water bridges (A_1_:w:N_1_) with respect to the interacting amino acids and ribonucleotides*

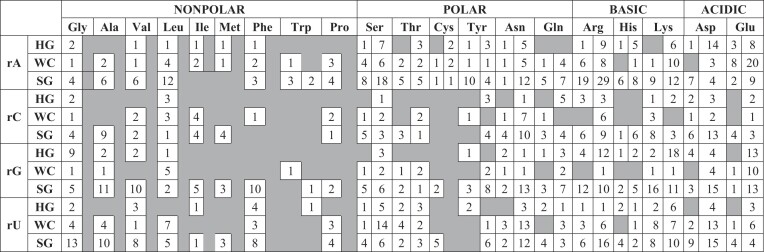

*First column under each amino acid corresponds to the main chain while the second column corresponds to side chain mediated water bridges.

**Table 2.** Frequency distribution of nucleobase-mediated cyclic triplet water bridges (*cyc*-A_1_:w:N_1_) with respect to interacting amino acids and ribonucleotide*

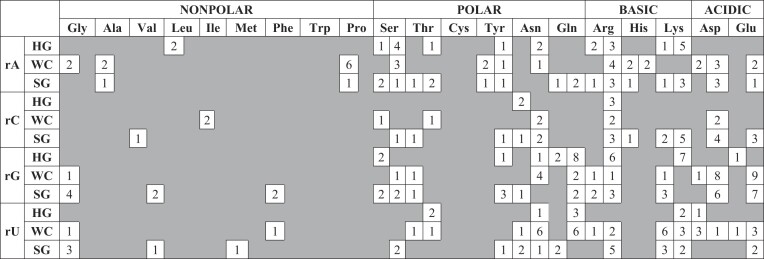

*First column under each amino acid corresponds to the main chain while the second column corresponds to side chain mediated water bridges.

456 (4 nucleobases × 3 nucleobase edges × 18 amino acids (all amino acid except Gly and Leu) × 2 amino acid interacting portions + 4 nucleobases × 3 nucleobase edges × 2 amino acids; Gly and Leu do not have any side chain portion capable of forming hydrogen bonds with water molecule) theoretical combinations exist for both A_1_:w:N_1_ and *cyc*-A_1_:w:N_1_ water bridges (Tables 1 and 2). Furthermore, due to their inherently greater flexibility compared to nucleobases, both the main chain and side chain entities of an amino acid can interact with the same bridging water. This results in an additional 216 theoretical combinations (4 nucleobases × 3 nucleobase edges × 18 amino acids × 1 (as both main chain and side chain are involved, Supplementary Tables S14 and S15). The 1279 acyclic A_1_:w:N_1_ motifs observed in crystal structures span 300 out of the 672 theoretical classes; 33 bridges involve both main chain and side chain of amino acids (Table 1 and Supplementary Table S14). In contrast, for the cyclic topology, the 289 observed bridges fall into only 133 of the 672 possible classes. This includes 12 water bridges that utilize both the main and side chains of an amino acid (Table 2 and Supplementary Table S15).

The most frequent composition in the A_1_:w:N_1_ topology involves the side chain of Arg and the sugar edge of rA (i.e. Arg(s):w:rA(SG)), and occurs in 29 cases (Figure 5A and Table 1). Similarly, within *cyc*-A_1_:w:N_1_, *cyc*-Glu(s):w:rG(WC) occurs most frequently (9 cases, Figure 5B and Table 2). It is worth noting that this nomenclature does not incorporate a sub-categorization for possible direct hydrogen bonds in the cyclic topologies, since this added level of organization will generate many gratuitous empty classes. Additionally, a high probability exists for the involvement of phosphate and ribose moieties in the formation of direct hydrogen bonds with amino acids, which will further create many classes, thus jeopardizing the simplicity of the classification scheme.

Our classification can also be applied to other higher-order water bridges, where additional amino acids and ribonucleotides can be separated by a delimiter (|). In order to provide unique systematic identifiers, all interacting amino acids are grouped according to their interacting portion (i.e. m, s or ms) and arranged in descending alphabetical order. Amino acids using their main chain are written to the immediate left of the central point of the nomenclature (:w:), followed by those which use both main chain and side chain, and then those using only their side chains. Similarly, ribonucleotides are arranged in alphabetical order and grouped by their interacting moiety (Nb, Ph, Rb), where the ribonucleotide interacting through the base moiety is specified first, followed by those interacting through phosphate, and finally those interacting through ribose. Within the group of ribonucleotides using nucleobase moieties, those employing their WC edge are written closest to the central point of the nomenclature (:w:), followed by those using the HG edge, then those using their SG edges respectively. For example, an A_3_:w:N_1_ water bridge can be notated as Tyr(s)|Ser(m)|Ala(m):w:rA(HG). This indicates that the main chains of Ala and Ser, the side chain of Tyr, and the HG edge of rA are involved in the formation of a quintet water bridge (Supplementary Figure S7A). Similarly, a sextet water bridge, which involves the phosphate moiety of rU, the ribose moiety of rC and rG, the main chain of Gly, and the side chain of Thr belonging to the cyclic A_2_:w:N_3_ topology, is described as *cyc*-Thr(s)|Gly(m):w:rU(Ph)|rC(Rb)|rG(Rb) (Supplementary Figure S9B).

## Discussion

Comparing the distribution of these water-mediated hydrogen bonds with the recently characterized direct hydrogen bonds in RNA–protein crystal structures (22) highlights some contrasting features (Table 3). First, the percentage contribution of amino acid side chain decreases from 71.7% in direct hydrogen bonds to 57.5% in water-bridged hydrogen bonds (Table 3 and Supplementary Table S6), suggesting that bridging water molecules empower the polar main chain of amino acids to interact with ribonucleotides (42.5%). This is consistent with the hydration of backbone residues more generally—the steric interference at the backbones makes these less likely to fold to bind to a residue, but a mediating water molecule alleviates this challenge. Second, the proportion of hydrogen bonds involving the main chain oxygen (63.3%) to that of nitrogen increases in the case of water bridges compared to direct hydrogen bonds (40.5%), suggesting a greater hydration of main chain oxygen atoms (Table 3). Third, the share of Arg in forming water-mediated hydrogen bonds decreases by almost 8% compared to direct hydrogen bonds (Table 3). Fourth, unlike in direct hydrogen bonds (22), the percent share of each nucleobase, ribose and phosphate moiety is almost equal as the ribose is more likely to participate in the case of water bridges than in direct hydrogen bonds. The hydroxyl groups of ribose favor water-mediated hydrogen bonds over direct ones. Fifth, the percentage contribution of Gly to hydrogen-bond formation with bridging water molecules is lowered by 5% than that of direct hydrogen bonds; this is likely not so much due to any preference toward Gly, but due to the less unhindered nature of Gly which makes direct bonds more feasible than for the other amino acids, although this less hindered nature is less important when interactions with the RNA are mediated by water. Sixth, although atom *N7* of rA does not participate in the formation of direct hydrogen bonds with the acidic groups of Asp and Glu due the difference between p*K*_a_ value of *N7* atom (rA) and the acidic side chains of Asp and Glu (44), surrounding water molecules can mediate this interaction. Indeed, we found 21 such water bridges involving the *N7* or adenosine and the carboxylates of Asp and Glu (Supplementary Table S16).

**Table 3.** Comparison of the metadata of hydrogen bonds involved in the formation of water bridges and direct hydrogen bonds between amino acids and ribonucleotides

Attribute	Present data (Hydrogen bonds with bridging water molecules)	Kagra *et al.* (22) (Direct hydrogen bonds excluding carbon mediated)
Number of crystal structures	329	293
Number of hydrogen bonds	6746	10 351
Purine %	57.6	54.4
Preferred nucleobase	rG (30.8%)	rG (32.4%)
% Base : Phosphate : Ribose	31.1 : 36.2 : 32.7	31.2 : 41.7 : 25.1
Preferred amino acid	Arg (17.8%)	Arg (25.8%)
% Side chain	57.5	71.7
% Polar amino acids	83.1	87.4
% Oxygen in all main chains	63.3	40.5

Very few statistical studies are available examining the nature of water bridges in RNA–protein complexes. This is due to the small dataset size, variable terminology and the lack of a tool to differentiate the topologies and the anatomy of the water bridges (16,17,44,46). For example, Treger *et al.* used a term ‘contacts through bridging water molecule’ to represent an interaction motif in which water forms hydrogen bonds with both a ribonucleotide and an amino acid (16). Such water-mediated interactions were called a ‘water-mediated bond’ in a subsequent study by Jeong *et al.* (17). However, Barik *et al.* confusingly used the term ‘water-mediated hydrogen bond’ to denote a single hydrogen bond with a water molecule by either an amino acid or a ribonucleotide, independent of whether the water was bridging or not (46). Moreover, existing terminologies do not consider the size of these interaction motifs. This is in contrast to this work where we clarify this by using the term ‘water bridge’ to describe a water-mediated interaction between at least one amino acid and one ribonucleotide with an adjective (triplet, quartet and so on) to define the size of the water bridge (Supplementary Figures S4–S9).

Treger *et al.* and Jeong *et al.* identified 309 and 1276 triplet water bridges respectively. However, these studies scaled down higher-order water bridges to a series of independent divalent water bridges (i.e. an A_2_:w:N_1_ water bridge was counted as two A_1_:w:N_1_ bridges) (16,17). This might group very different systems into the same category. We removed this redundancy by considering the order of water bridges and identified a total of 4963 unique water bridges of different orders, out of which 2372 correspond to the definition of Treger *et al.* and Jeong *et al.* (16,17). Barik *et al.* presented a general statistical study of 2440 interfacial water molecules involving 878 bridging waters, although comparison is impossible as we only focus on bridging waters that help in RNA–protein interaction, whereas Barik *et al.* considered protein–protein and RNA–RNA bridges as well (46). This is a very valid choice, but then one might consider any exploration of those datasets to necessarily include the rest of the PDB where only proteins or nucleic acids occur, as it is not clear why an amino acid–amino acid water bridge in an RNA–protein structure would be meaningfully different than an amino acid–amino acid water bridge in a quaternary protein structure, or even in a pocket of a single polypeptide. As there are many more examples of these in the PDB than RNA–protein structures, any analysis from this current data subset would be only partial at best. Consequently, we focused only on the bridges between the classes of biomacromolecules and excluded the within-class water bridges from our analysis.

Previously, Kondo *et al.* identified eight different geometries of hydrogen-bonded *pseudo* pairs between nucleotides and five amino acids (Asn, Gln, Asp, Glu and Arg) in the crystal structures of nucleotide–protein complexes. These all fit within our definition of *cyc*-A_1_:w:N_1_ water bridges (44). In total, we identified 125 unique geometries for the *cyc*-A_1_:w:N_1_ water bridges including at least one example involving each of the 20 canonical amino acids, 59 of which involve Asn, Gln, Asp, Glu and Arg. Furthermore, in addition to expanding the repertoire of triplet geometries of water bridges identified by Kondo *et al.* (44), we identified 1270 total examples of cyclic water bridges involving different amino acids, which helps improve our understanding of molecular recognition. We further present a viable classification scheme for cyclic triplet water bridges (522 examples and 720 unique classes), which can be directly applied to the water-mediated *pseudo* pairs identified by Kondo *et al.* (44). However, dissenting from Kondo *et al.*, we find a significant participation of the SG edges in *cyc*-A_1_:w:N_1_ water bridges. In addition, although Kondo *et al.* found only one example of water-mediated *pseudo* base pair between the acidic group of Asp or Glu and the HG edge of adenine (i.e. *cyc*-Asp(s):w:rA(HG) or *cyc*-Glu(s):w:rA(HG)), we identified an extended version of Kondo *et al.*’s *cyc*-Asp(s):w:rA(HG) triplet belonging to the *cyc*-A_2_:w:N_1_ topology (i.e*. cyc*-Asp(s)|Lys(s):w:rA(HG)). Indeed, such extended interaction motifs better describe the surrounding structural environment within the macromolecular context and thereby impart precision and specificity to the understanding of macromolecular recognition.

Interestingly, Foss *et al.* reported the change in the water-mediated network of hydrogen bonds on replacement of even a single amino acid residue in the p19 protein in complexation with siRNA, which in turn is responsible for alterations in the enthalpy of RNA binding (59). This change in enthalpy can be considered as a sum of contributions from the changes in direct hydrogen bonds and water bridges. Extending this argument, we speculate that each water bridge has its own characteristic contribution to the enthalpy of RNA–protein complex. Therefore, our classification of water bridges in combination with quantum chemical calculations can help estimate the contribution of each class of water bridges toward the enthalpy of RNA:protein binding. Providing typical quantum chemically calculated stabilities or interaction energies for each of these bridges to conduct these analyses would be a useful extension of this work but lies beyond the scope of the current study.

## Conclusion

A total of 4963 RNA–protein water bridges were identified in 329 high-resolution crystal structures of RNA–protein complexes. The structure and distribution of these water bridges is complex and highly diverse. To establish their physiochemical roles and offer greater understating of their contribution toward molecular recognition, we present a graph theory-based classification scheme, in which water bridges are presented as multiplets. According to this scheme, the simplest water bridges are triplets—i.e. A_1_:w:N_1_—and are the most frequent.

As the structural size and order of the topology increases, the frequency of that topology decreases (Figure 6A). This is consistent with many statements around complexity or assembly theory (60). Higher-ordered topologies are complex, where water is present in a more confined space and is less dynamic. This observation is confirmed by the B-factor analysis of water molecules. The existence of higher topologies hints toward more specific roles played by water molecules and will intrigue researchers to carefully observe such substructures while framing rules of molecular recognition and prediction of hydration patterns.

The proposed classification and analysis can be used in combination with quantum chemical studies and molecular dynamics simulations to craft precise machine learning models for the identification of druggable pocket at the RNA-binding interfaces. This can in turn open novel avenues for the prediction of hydration patterns and molecular docking, and may also be useful for developing the interaction network diagrams for RNA–protein complexes, as proposed for RNA by Lescoute *et al.* (61). This theoretical framework can also be adapted to study water bridges connecting amino acid entities of two proteins at the protein-protein interface.

Beyond cataloging these interactions, our study underscores their functional significance. By elucidating the diverse roles of water as an organizing principle in biomolecular interactions, our findings contribute to understanding the nuanced dynamics within RNA–protein assemblies. Moreover, our work invites exploration into the high multiplicity spaces where these water-mediated interactions may play pivotal roles. These insights are pivotal not only for comprehending existing biomolecules but also for informing the rational design of novel RNA–protein complexes.

In conclusion, this study advances the fundamental understanding of water-mediated interactions in RNA–protein complexes by revealing preferential binding patterns and highlighting the water-based structural and functional relationships between ribonucleotides and amino acids in biomolecular complexes, thereby offering a foundational resource for further research into their functional relevance and potential applications in biomolecular design and therapeutics.

## Supplementary Material

lqae161_Supplemental_Files

## Data Availability

The data supporting this article are available in the article itself, in its online supplementary information files and on GitHub (https://github.com/PSCPU/waterbridges) as well as Figshare (https://doi.org/10.6084/m9.figshare.26380246.v3).
