# Supplementary material for: Water-mediated ribonucleotide–amino acid pairs and higher-order structures at the RNA–protein interface: analysis of the crystal structure database and a topological classification
Source: NAR Genom Bioinform. 2024 Dec 11;6(4):lqae161. doi: 10.1093/nargab/lqae161 (PMC11632616; doi:10.1093/nargab/lqae161)
Supplement: lqae161_Supplemental_Files [file lqae161_supplemental_files.zip › Water bridges - Supplementary Information.pdf]

Electronic Supplementary Information (ESI)

For

Water-mediated ribonucleotide–amino acid pairs and higher order structures at the RNA–protein interface: Analysis of the crystal structure database and a topological classification

Raman Jangra[1], John F. Trant[2,3,4,*] and Purshotam Sharma[1,2,*]

[1]Computational Biochemistry Laboratory, Department of Chemistry and Centre for Advanced Studies in Chemistry, Panjab University, Chandigarh, 160014, India.
[2]Department of Chemistry and Biochemistry, University of Windsor, 401 Sunset Ave. Windsor, ON, N9B 3P4, Canada
[3]We-Spark Health Institute, 401 Sunset Ave. Windsor ON, N9B 3P4, Canada
[4]Binary Star Research Services, LaSalle, ON, N9J 3X8, Canada
*Email: psharma@pu.ac.in, psharma@uwindsor.ca, j.trant@uwindsor.ca

TABLE OF CONTENTS

Figure S1: Role of water-mediated hydrogen bonding interactions…………..…………...SI-3
Figure S2. Chemical structure of canonical ribonucleotides...……...…......………………SI-4
Figure S3. Chemical structure of amino acid residue..………………..……………………SI-5
Figure S4: Examples of quartet water bridges (A_1:w:N_2)………….. ……………………SI-6
Figure S5: Examples of quartet water bridges (A_2:w:N_1)…………………….……………SI-7
Figure S6: Examples of quintet water bridges (A_1:w:N_3)…………………..………………SI-8
Figure S7: Examples of quintet water bridges (A_3:w:N_1)……………..……………………SI-9
Figure S8: Examples of quintet water bridges (A_2:w:N_2)………………... ……………...SI-10
Figure S9: Examples of the highest-identified topology…..……...………………………SI-11
Table S1. PDB codes of analyzed 329 crystal structures of RNA–protein complexes.…...SI-12
Table S2. The list of atoms of nucleobases interacting independently creates ambiguity while defining the interacting edge of those nucleobases……………………………..……SI-13
Table S3. Frequency of RNA types present in the 329 analyzed crystal structures of RNA–protein complexes……..……………………………………………..………………SI-14
Table S4. Frequency of different types of proteins present in the 329 crystal structures of RNA–protein complexes……………………………………………………………..…SI-15
Table S5: Distribution of bridging water molecules in terms of hydrogen bonds…....…..SI-16
Table S6: Counts of amino acid residues involved in water bridge formation…..….……SI-17
Table S7: Counts of ribonucleotides involved in water bridge formation…………….…SI-18
Table S8: Counts of ribonucleotides' atoms involved in water bridge formation……..…SI-19
Table S9: Counts of atoms of amino acid residues involved in water bridge formation…SI-20
Table S10: Counts of different topologies of water bridges………………..………..…SI-21
Table S11: Counts of ribonucleotide moieties involved in triplet and other higher-ordered topologies……………………………..…………………………………..…SI-22
Table S12: Counts of amino acid portions involved in triplet and other higher-ordered topologies……………………………………..………………………………...SI-23
Table S13: Average B-factors of different identified topologies……...………………..SI-24
Table S14: Frequency distribution of nucleobase-mediated A_1:w:N_1 water bridges……...SI-25
Table S15: Frequency distribution of nucleobase-mediated *cyc*-A_1:w:N_1 water bridges…SI-26
Table S16: Water bridges using *N7* (rA) to interact with acidic groups of Asp and Glu…SI-27
References..……………………………………………………………………..SI-28

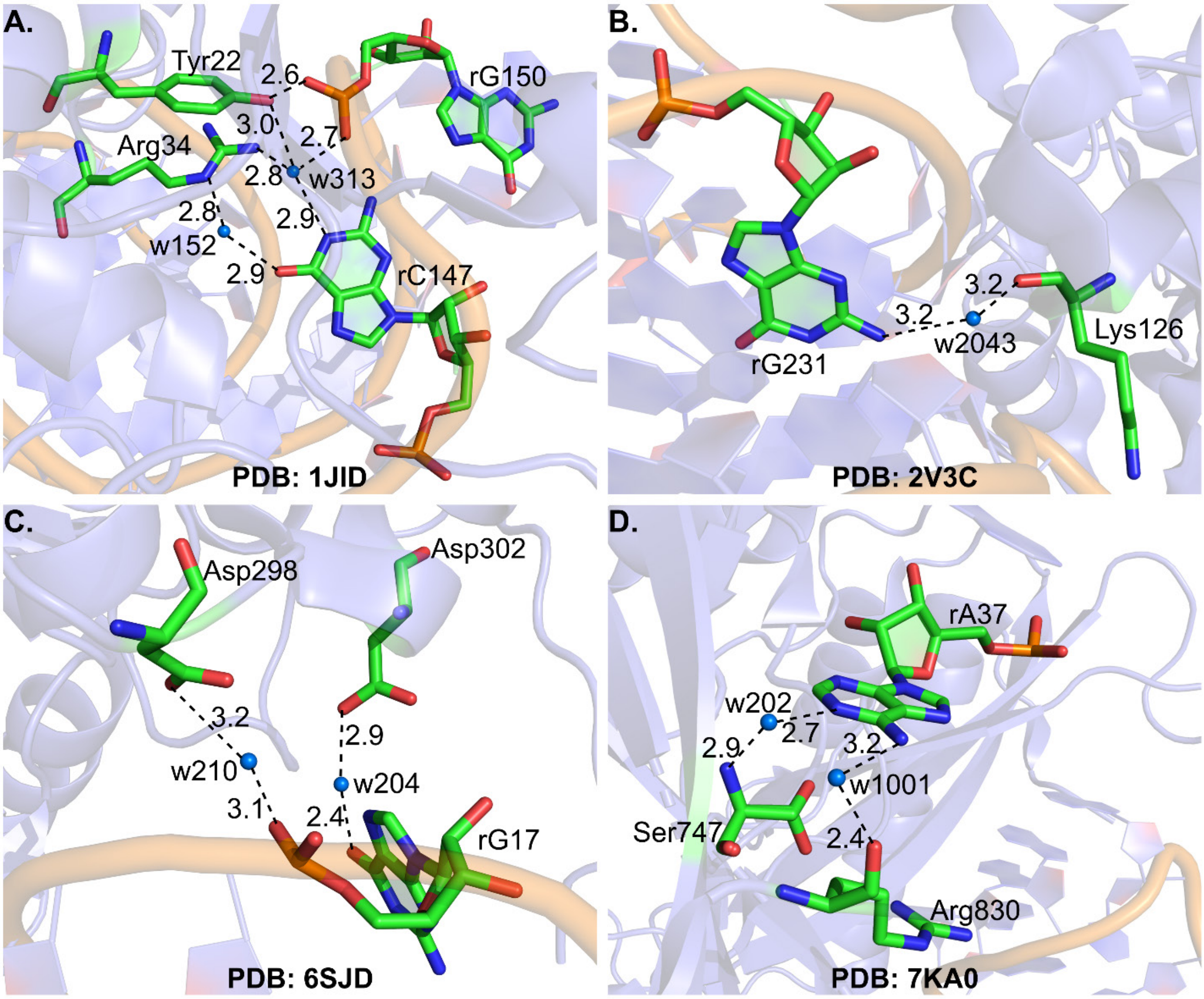

Figure S1. Water–mediated interactions facilitate the specific binding of: A. Human signal recognition particle 19 (SRP19) to SRP RNA(1); B. *Methanococcus jannaschii* SRP54 to SRP RNA(2); C. Cytoplasmic protein ZC3H12B to RNA(3); & D. *Mycobacterium tuberculosis* Phe-tRNA synthetase to $tRNA^{Phe}$(4). Water molecules (w) are represented by marine-blue spheres. Donor-acceptor (D–A) distances (Å) are provided for each water-mediated interaction.

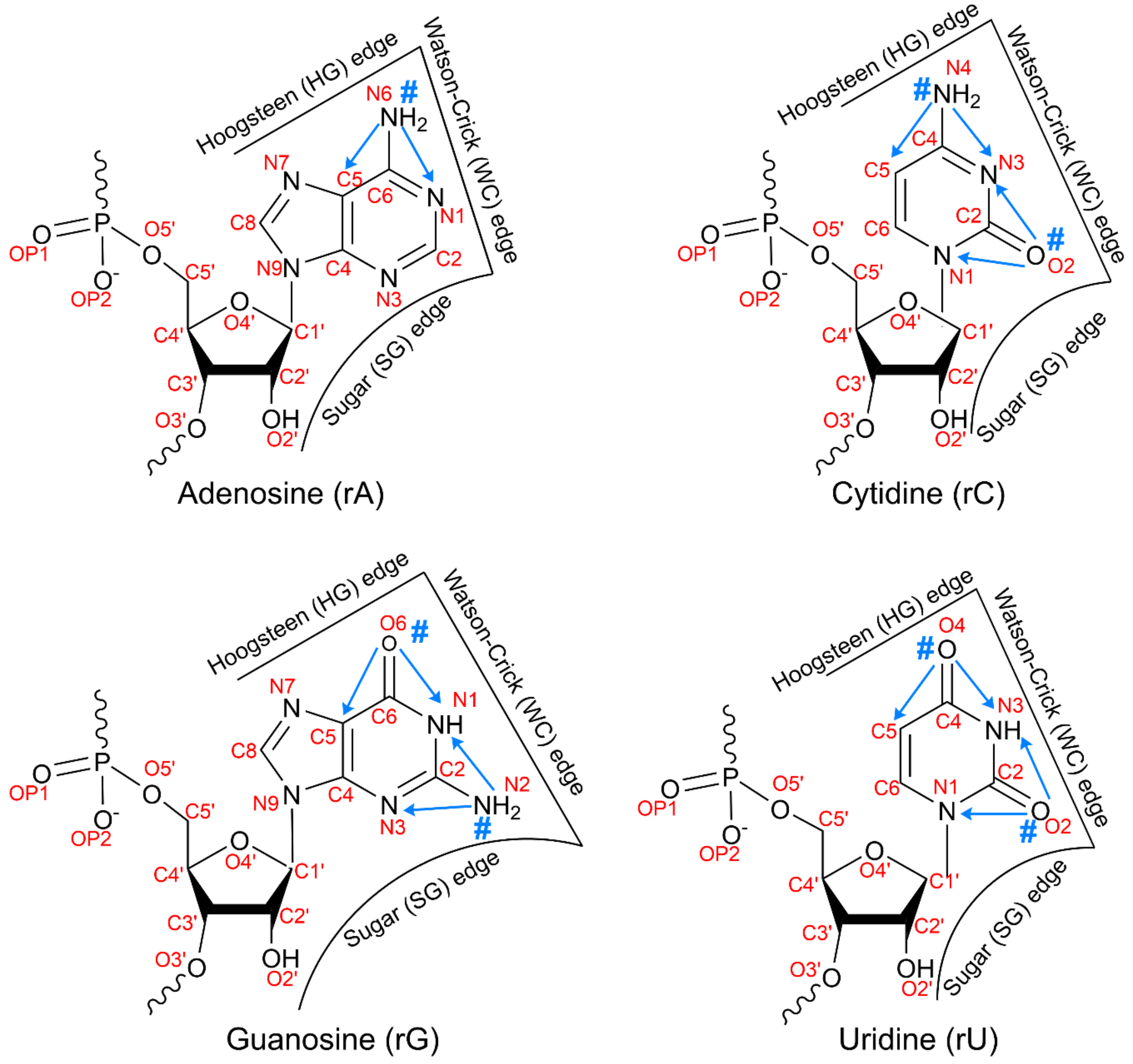

Figure S2. Chemical structures of the four canonical ribonucleotides, along with their atom names in PDB files (red). Wavy bonds represent the points of attachment of the ribonucleotides to the rest of the RNA chain. Blue hashes (#) indicate the atoms which cause ambiguity in the classification of the interacting edges, and arrows point toward the atoms from which distance to the interacting water molecule was additionally calculated for determining which face was interacting when the hashed atoms were interacting through a hydrogen bond.

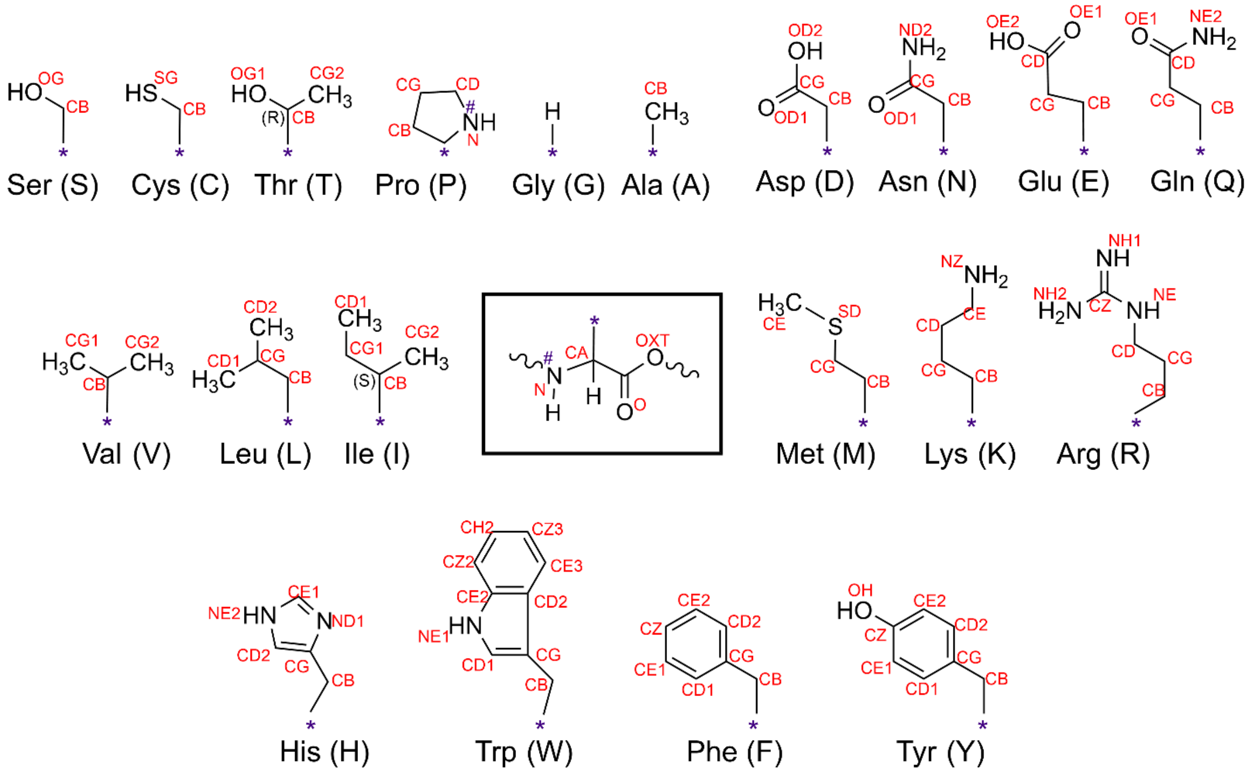

Figure S3. Chemical structures of the 20 amino acid residues along with their atom names in PDB files (red). The main chain of amino acids is shown in a box with their corresponding side chains on the periphery. Wavy bonds represent the points of attachment of the next amino acid residue to the polypeptide chain. Asterisk (*) represents the point of attachment of side chain to the main chain. The stereochemistry of the chiral side chain carbon of Thr and Ile is mentioned.

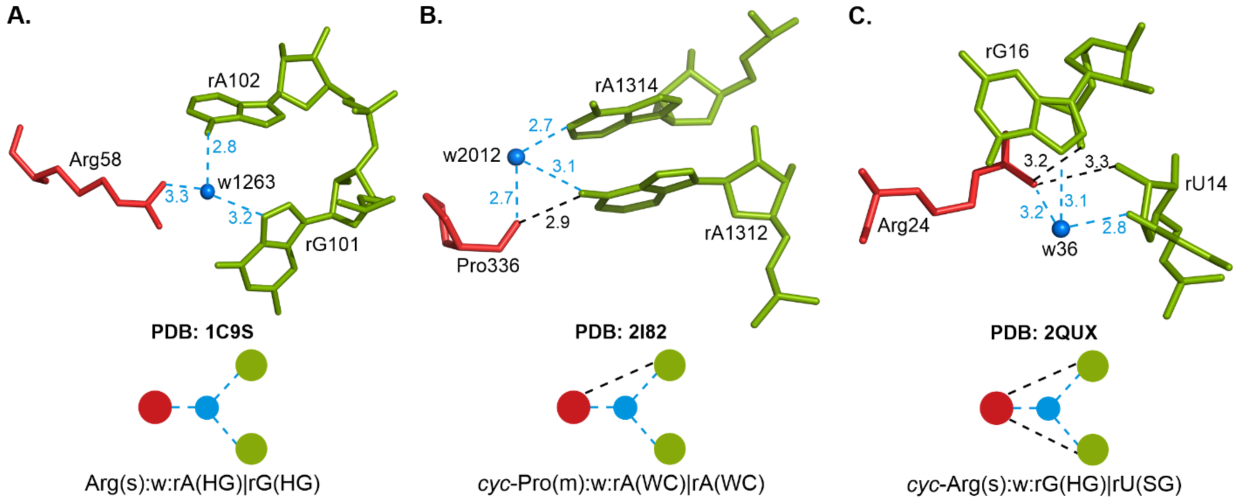

Figure S4. Examples of A_1:w:N_2 quartet water bridges, where water molecule is predicated to facilitates the specific binding of A. *trp* RNA-binding attenuation protein (TRAP) with a 53-base single stranded RNA (5), B. Ribosomal large subunit pseudouridine synthase A (RluA) with an anticodon stem-loop (ASL) RNA (6), and C. *Pseudomonas aeruginosa* bacteriophage PP7 coat protein with its translational operator RNA (7) along with their topological representation and nomenclature. Amino acids and their corresponding nodes are highlighted in red while ribonucleotides and their corresponding nodes are highlighted in green. Water molecules are represented in blue. Blue vertices/dashed lines indicate water bridges, while black vertices represent direct hydrogen bonds between amino acids and ribonucleotides. Donor-acceptor (D–A) distances (in Å) are provided for each interaction.

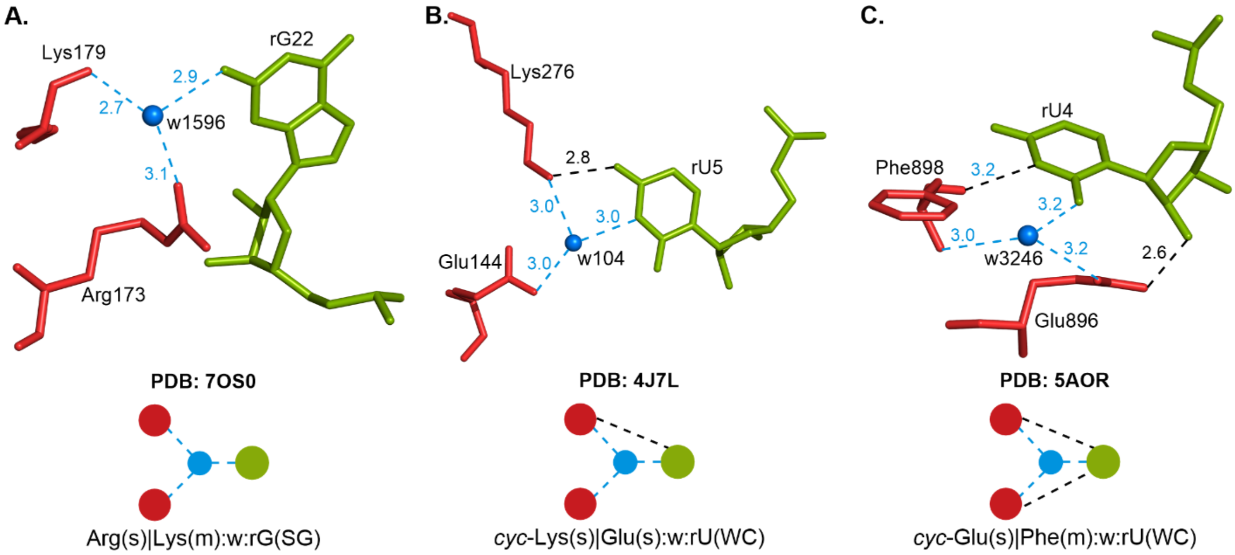

Figure S5. Examples of A_2:w:N_1 quartet water bridges, where water molecule mediates specific binding of A. Cas13a enzyme from the bacteria *Rhodobacter capsulatus* to crRNA (8), B. Protein Dom3Z (a mammalian homolog of yeast proteins, Rai1 and Dxo1) to pre-mRNA (9), and C. dosage compensation regulator, *Drosophila* MLE helicase to RNA (10) along with their topological representation and nomenclature. Amino acids and their corresponding nodes are highlighted in red while ribonucleotides and their corresponding nodes are highlighted in green. Water molecules are represented in blue. Blue vertices/dashed lines indicate water bridges, while black vertices represent direct hydrogen bonds between amino acids and ribonucleotides. Donor-acceptor (D–A) distances (in Å) are provided for each interaction.

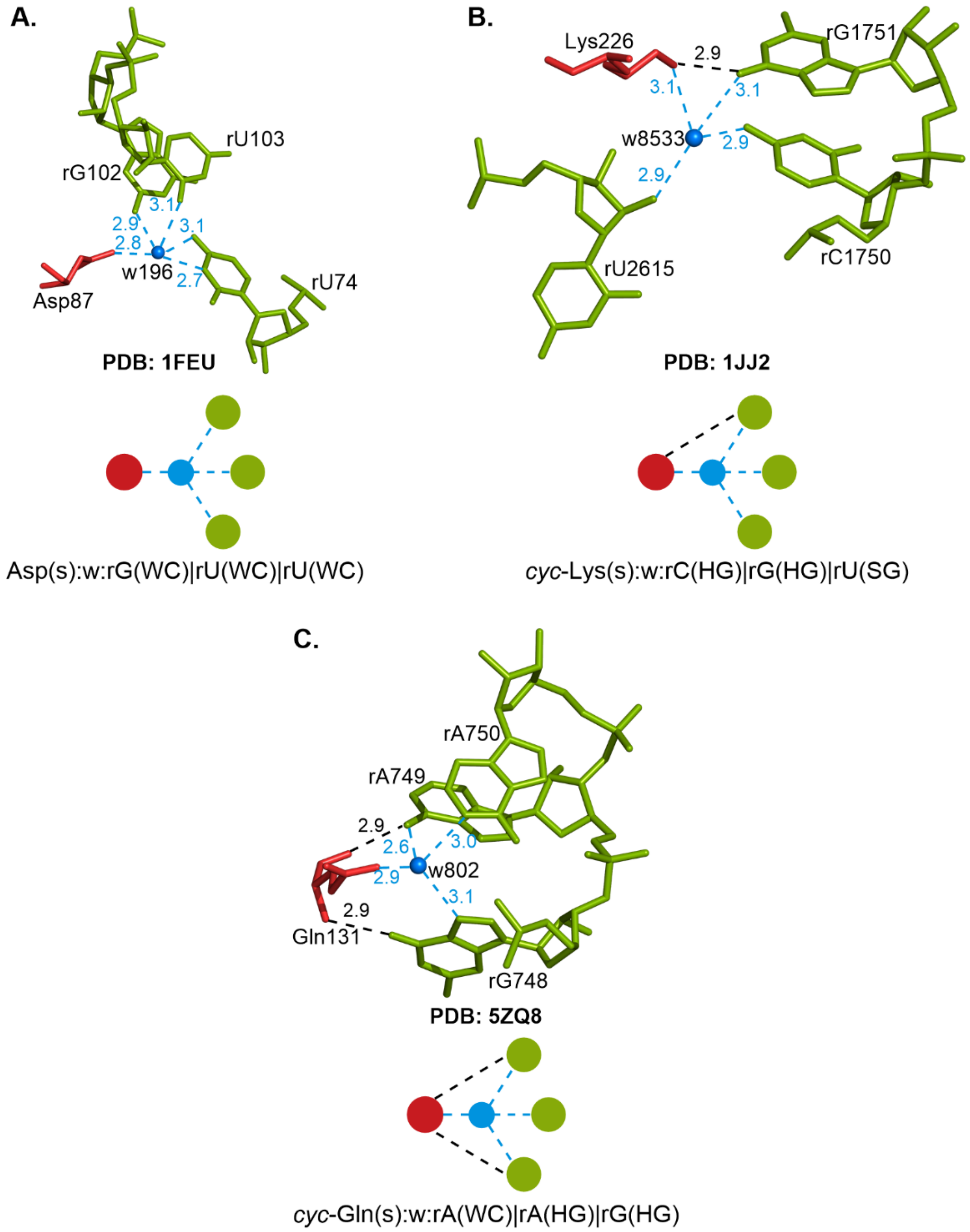

Figure S6. Examples of A_1:w:N_3 quintet water bridges, where water molecule is expected to assist specific recognition of A. 19 nt fragment of *Escherichia coli* 5S rRNA by *Thermus thermophilus* ribosomal protein TL5 (11), B. *Haloarcula marismortui* 23S rRNA by ribosomal protein L3 (12), and C. *Streptococcus pneumonia* 23S rRNA by methyltransferase RlmCD (13), along with their topological representation and nomenclature. Amino acids and their corresponding nodes are highlighted in red while ribonucleotides and their corresponding nodes are highlighted in green. Water molecules are represented in blue. Blue vertices/dashed lines indicate water bridges, while black vertices represent direct hydrogen bonds between amino acids and ribonucleotides. Donor-acceptor (D–A) distances (in Å) are provided for each interaction.

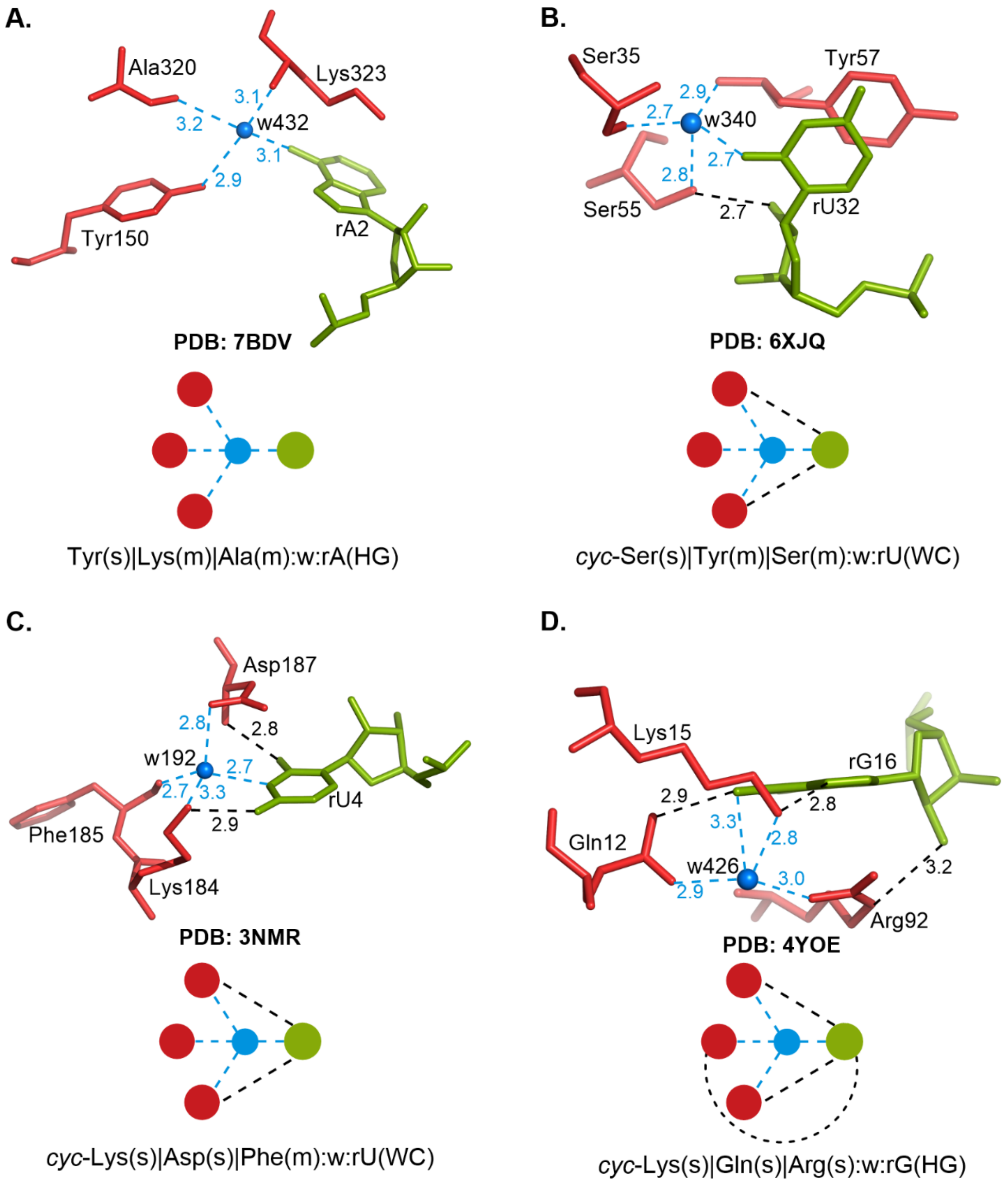

Figure S7. Examples of A_3:w:N_1 quintet water bridges, where water molecule assists in specific binding of A. CRISPR ancillary nuclease 2 (Can2) protein with cyclic tetraadenosine monophosphate (cA4) (14), B. fragment antigen-binding (Fab) of Hepatitis A virus (HAV) with self-alkylating 58-mer ribozyme (15), C. Human CUG-binding protein 1 (CUGBP1) with RNA (16), and D. heterogeneous nuclear ribonucleoprotein (hnRNP) A1 protein with a 5'-AGU-3' trinucleotide (17) along with their topological representation and nomenclature. Amino acids and their corresponding nodes are highlighted in red while ribonucleotides and their corresponding nodes are highlighted in green. Water molecules are represented in blue. Blue vertices/dashed lines indicate water bridges, while black vertices represent direct hydrogen bonds between amino acids and ribonucleotides. Donor-acceptor (D–A) distances (in Å) are provided for each interaction.

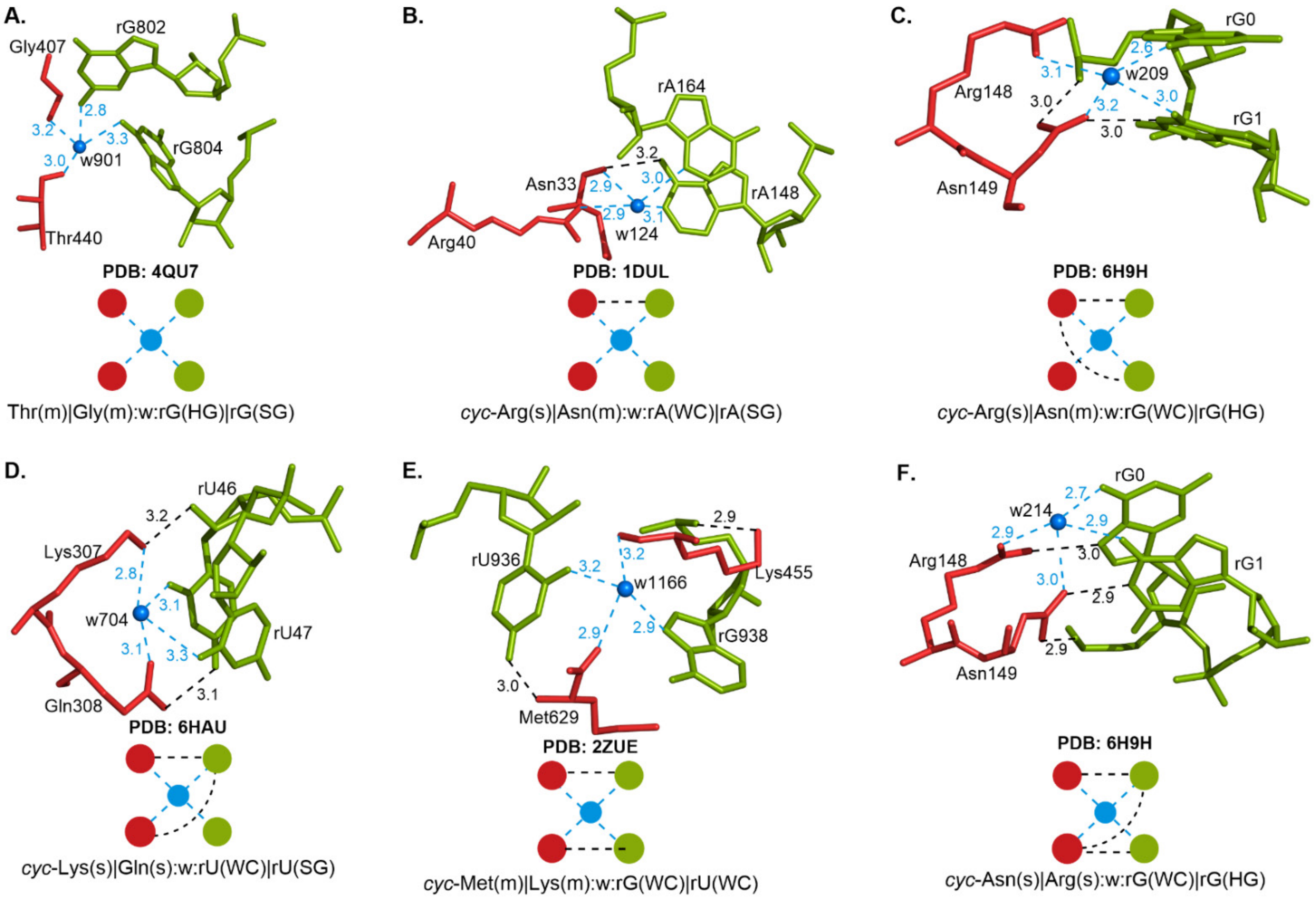

Figure S8. Examples of A_2:w:N_2 type quintet water bridges, where water molecule assists in specific binding of A. human G-rich RNA sequence binding factor 1 (GRSF1) with RNA, B. signal recognition particle (SRP) protein with 4.5S RNA (18), C. Csf5 protein with *aromatoleum aromaticum* EbN1 crRNA (19), D. Kaposi's sarcoma-associated herpesvirus (KSHV) ORF57-Mta protein with Mta responsive element of polyadenylated nuclear (PAN) RNA (20), E. *Pyrococcus horikoshii* arginyl-tRNA synthetase with $tRNA^{Arg}$ (21), and F. Csf5 protein with *aromatoleum aromaticum* EbN1 crRNA (19), along with their topological representation and nomenclature. Amino acids and their corresponding nodes are highlighted in red while ribonucleotides and their corresponding nodes are highlighted in green. Water molecules are represented in blue. Blue vertices/dashed lines indicate water bridges, while black vertices represent direct hydrogen bonds between amino acids and ribonucleotides. Donor-acceptor (D–A) distances (in Å) are provided for each interaction.

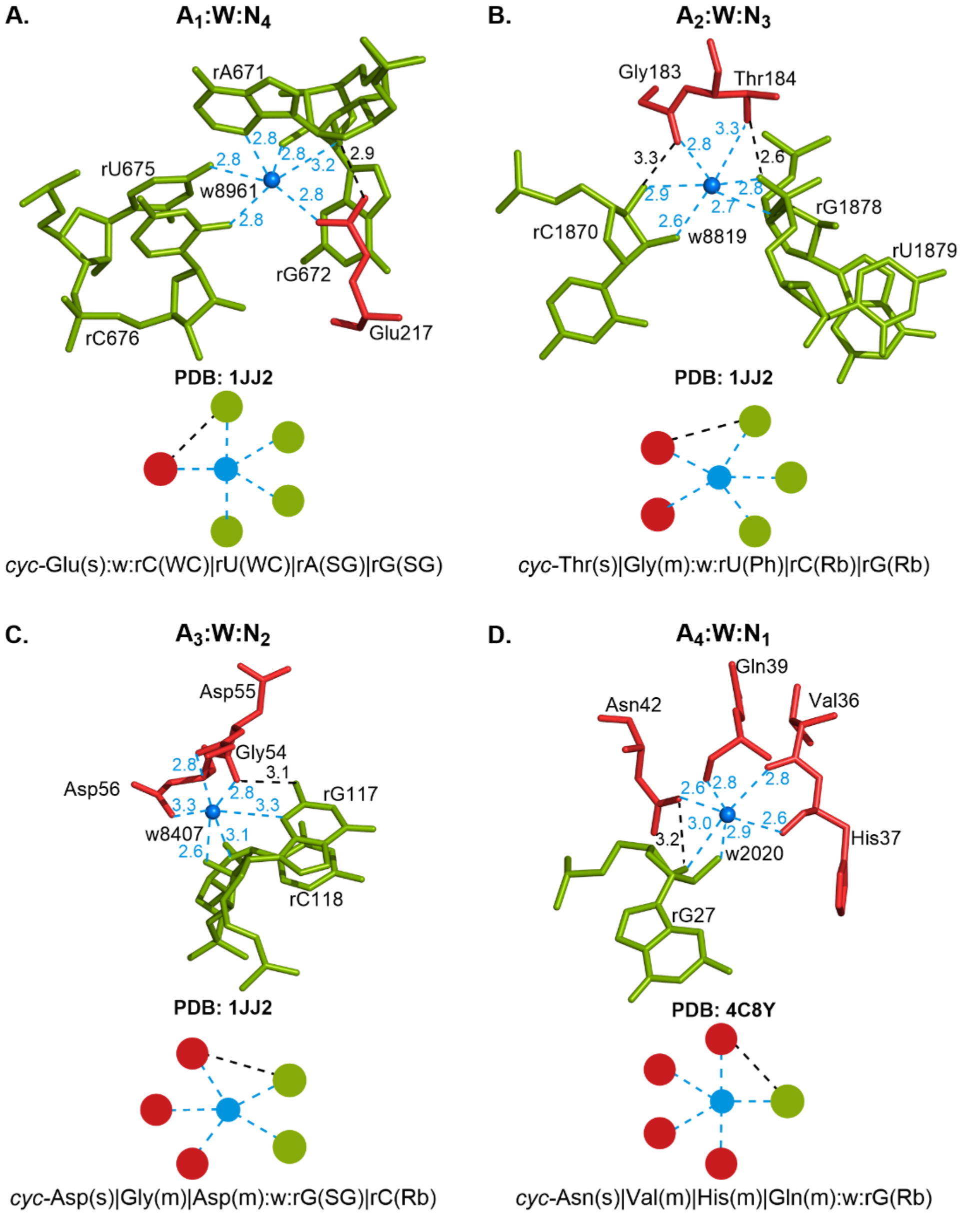

Figure S9. Examples of sextet water bridges, the highest order topology identified in RNA:protein complexes along with their topological representation and nomenclature. A. A_1:w:N_4 topology formed between *Haloarcula marismortui* 23S rRNA and ribosomal protein L4 (12), B. A_2:w:N_3 topology formed between *Haloarcula marismortui* 23S rRNA and ribosomal protein L2 (12), C. A_3:w:N_2 topology formed between *Haloarcula marismortui* 5S rRNA and ribosomal protein L18 (12), and D. A_4:w:N_1 topology formed between CRISPR-associated 6A (Cas6A) endonucleases and R1 RNA (22). Amino acids are represented in red while ribonucleotides are represented in green. Water molecules are represented in blue. Blue vertices/dashed lines indicate water bridges, while black vertices represent direct hydrogen bonds between amino acids and ribonucleotides. Donor-acceptor (D–A) distances (in Å) are provided for each interaction.

Table S1. PDB codes for the 329 crystal structures of RNA–protein complexes included in our dataset. The number of bridging water molecules and the total number of water molecules present in each crystal structure are provided in parentheses.

1A34 (3, 168), 1B2M (1, 92), 1C0A (19, 514), 1C9S (30, 1264), 1DFU (10, 242), 1DUL (8, 299), 1EC6 (7, 170), 1F7U (26, 588), 1FEU (11, 295), 1FFK (0, 6), 1FFY (16, 328), 1G59 (4, 272), 1GTF (18, 1466), 1GTN (0, 73), 1J1U (4, 364), 1JBR (7, 168), 1JID (9, 277), 1JJ2 (887, 7893), 1K8W (21, 259), 1KNZ (22, 605), 1LNG (13, 208), 1M5K (9, 103), 1MJI (9, 187), 1N35 (7, 350), 1O0B (22, 144), 1OOA (11, 294), 1R3E (13, 263), 1RPU (7, 111), 1SDS (13, 273), 1SJ3 (5, 37), 1U0B (11, 104), 1UVI (2, 418), 1WMQ (7, 314), 1YTU (2, 57), 1YVP (39, 408), 1YYK (1, 188), 1ZH5 (22, 455), 2A8V (0, 81), 2ANN (3, 77), 2ASB (7, 260), 2B3J (18, 300), 2BH2 (24, 340), 2BS0 (0, 188), 2DB3 (22, 1319), 2DLC (1, 57), 2F8K (4, 117), 2G4B (3, 30), 2GXB (8, 157), 2HVY (3, 118), 2HYI (13, 660), 2I82 (40, 255), 2J0S (4, 344), 2JEA (4, 122), 2JLU (12, 407), 2NZ4 (0, 206), 2PJP (10, 112), 2PO1 (6, 308), 2Q66 (13, 509), 2QUX (14, 512), 2R8S (16, 525), 2RD2 (15, 136), 2UWM (18, 292), 2V3C (9, 474), 2VNU (11, 450), 2VPL (16, 318), 2X1A (2, 44), 2XLI (1, 33), 2XNR (1, 47), 2XS2 (12, 129), 2XZL (5, 96), 2XZO (7, 125), 2ZKO (27, 205), 2ZUE (17, 362), 3ADD (3, 160), 3ADL (2, 47), 3AGV (8, 253), 3AHU (0, 25), 3AM1 (1, 7), 3AVX (4, 105), 3B0U (8, 418), 3BSN (23, 347), 3BT7 (16, 261), 3D2S (11, 384), 3DD2 (10, 253), 3EGZ (7, 146), 3EQT (10, 215), 3ER9 (1, 317), 3EX7 (8, 380), 3FHT (9, 300), 3FOZ (5, 73), 3G9Y (5, 31), 3GIB (3, 9), 3GPQ (5, 250), 3HJW (10, 99), 3IEM (0, 224), 3IEV (8, 245), 3K49 (6, 431), 3KS8 (13, 150), 3M7N (16, 716), 3MDG (4, 206), 3MJ0 (2, 33), 3MXH (5, 169), 3NMR (5, 96), 3NNC (5, 63), 3O7V (4, 52), 3O8C (9, 796), 3OIN (7, 241), 3OVB (29, 475), 3PEW (6, 524), 3PF4 (5, 190), 3Q0Q (6, 206), 3QG9 (14, 290), 3QRP (14, 152), 3QSU (0, 207), 3R2D (8, 98), 3R9W (6, 91), 3RW6 (16, 135), 3SQW (5, 187), 3T3O (0, 97), 3T5N (10, 184), 3TS2 (29, 311), 3U2E (8, 150), 3U4M (10, 203), 3VJR (7, 139), 3VYX (6, 82), 3WBM (14, 310), 3ZGZ (19, 369), 4AFY (5, 268), 4AL5 (7, 128), 4ATO (15, 222), 4BPB (7, 107), 4BW0 (5, 53), 4C8Y (18, 380), 4D25 (5, 330), 4ED5 (23, 251), 4F02 (19, 196), 4G0A (13, 1155), 4GHA (18, 156), 4GV3 (6, 177), 4H5P (52, 545), 4HOR (12, 425), 4I67 (0, 11), 4II9 (2, 296), 4ILL (0, 12), 4IQX (4, 41), 4J7L (5, 365), 4JNG (13, 163), 4JZU (0, 219), 4K4X (75, 729), 4KZD (13, 466), 4L8H (1, 90), 4LGT (49, 630), 4LJ0 (4, 25), 4M4O (8, 220), 4M59 (12, 114), 4MDX (6, 354), 4N0T (40, 696), 4NGB (2, 194), 4NKU (10, 356), 4O8J (11, 610), 4OAV (22, 911), 4OHY (1, 148), 4OOG (12, 98), 4PKD (16, 113), 4PR6 (5, 83), 4QIK (14, 885), 4QM6 (25, 559), 4QOZ (22, 297), 4QU6 (4, 83), 4QU7 (4, 182), 4R3I (5, 87), 4R8I (0, 111), 4RCJ (4, 175), 4RCM (0, 97), 4RMO (109, 1322), 4RWN (17, 253), 4S2X (2, 109), 4S3N (17, 292), 4WAL (5, 83), 4WTJ (8, 377), 4X4T (10, 357), 4XWW (21, 759), 4YCO (19, 613), 4YOE (2, 90), 4YYE (31, 636), 4Z0C (18, 361), 4ZDO (2, 469), 4ZLR (8, 338), 5A0T (4, 558), 5AH5 (35, 871), 5AOR (21, 599), 5AOX (23, 381), 5AXM (3, 102), 5BTE (11, 357), 5BUD (17, 1044), 5BZ1 (11, 208), 5C0Y (12, 588), 5CCB (10, 416), 5D0A (6, 794), 5DDP (11, 321), 5DET (6, 135), 5DO4 (8, 163), 5ELH (8, 307), 5ELK (0, 19), 5ELT (1, 11), 5F8H (10, 141), 5GUH (2, 84), 5GXH (9, 317), 5HAB (10, 282), 5HJZ (8, 150), 5HK0 (1, 172), 5HR7 (21, 94), 5I4A (19, 704), 5I9F (15, 76), 5ID6 (0, 6), 5JAJ (36, 563), 5JJU (0, 166), 5JRC (3, 218), 5JS1 (5, 52), 5K77 (14, 518), 5L2L (58, 685), 5M0I (18, 333), 5M3H (18, 250), 5N94 (3, 67), 5O1Y (3, 38), 5O58 (7, 279), 5SZE (0, 36), 5THE (40, 1423), 5UD5 (6, 24), 5UDI (12, 394), 5VOE (0, 159), 5W0M (19, 205), 5W1H (86, 812), 5W3V (23, 250), 5WLH (77, 765), 5WWW (8, 93), 5WWX (6, 62), 5WZH (1, 24), 5XTM (17, 306), 5YKI (12, 144), 5ZC9 (7, 246), 5ZQ8 (6, 67), 5ZSA (2, 216), 5ZUU (9, 217), 5ZW4 (0, 162), 6A4E (2, 62), 6A6J (4, 51), 6D12 (0, 46), 6D1V (5, 288), 6D2Z (5, 161), 6DB8 (10, 252), 6DCB (10, 149), 6DTD (44, 866), 6DU4 (28, 385), 6E0O (1, 377), 6E4P (10, 314), 6EEN (28, 214), 6F4G (39, 869), 6FPX (8, 220), 6FQ3 (8, 237), 6FQR (6, 298), 6GC5 (17, 191), 6H9H (38, 307), 6HAU (6, 431), 6I0T (4, 91), 6IJ2 (21, 613), 6IS0 (0, 313), 6IV8 (76, 665), 6JIM (7, 352), 6JVX (7, 127), 6L5N (2, 156), 6LSH (6, 103), 6M6R (5, 124), 6M7K (9, 537), 6MFN (0, 87), 6N6A (6, 301), 6N6I (11, 396), 6O5F (2, 72), 6O6V (2, 22), 6O7B (2, 28), 6OON (6, 464), 6P7P (13, 861), 6PPN (3, 94), 6PPQ (7, 302), 6PUN (8, 166), 6RTI (10, 399), 6S0M (2, 343), 6SCE (13, 410), 6SCF (33, 589), 6SX0 (26, 160), 6TQB (18, 227), 6U6Y (5, 209), 6U8D (11, 386), 6UEJ (5, 93), 6UV1 (6, 550), 6VM6 (12, 1490), 6VRD (12, 350), 6WXQ (9, 284), 6WXY (0, 218), 6X1B (2, 432), 6X5M (3, 65), 6XJQ (36, 835), 6XLV (17, 268), 6YRB (2, 55), 6YUD (44, 573), 6YWO (33, 999), 6ZM2 (13, 194), 7A9X (18, 79), 7BDV (13, 271), 7C45 (16, 225), 7CGF (14, 204), 7D8O (100, 498), 7DIC (3, 128), 7JYY (10, 487), 7K5L (2, 99), 7K98 (30, 667), 7K9D (9, 240), 7KFN (7, 252), 7KL3 (2, 120), 7MJV (2, 37), 7MLX (8, 179), 7MPL (62, 1866), 7NDJ (1, 376), 7NJC (1, 61), 7OM6 (29, 555), 7OS0 (70, 630), 7OZQ (31, 597), 7R9G (2, 50), 7S02 (7, 125).

Table S2. List of nucleobase atoms involved in the ambiguous assignment of nucleobase interacting edges.

**Nucleobase**	**Watson-Crick (WC) edge**	**Hoogsteen (HG) edge**	**Sugar (SG) edge**
**A**	*N6*, *N1*	*N6*, *N7*	*N3*
**G**	*O6*, *N1*, *N2*	*O6*, *N7*	*N2*, *N3*
**C**	*N4*, *N3*, *O2*	*N4*	*O2*
**U**	*O4*, *N3*, *O2*	*O4*	*O2*

Table S3. Frequency of different RNA types present in the 329 analyzed crystal structures of RNA–protein complexes.

**RNA type**	**# of structures**	**PDB codes**	**Total # of water bridges**
ssRNA	130	1B2M, 1C9S, 1SDS, 1UVI, 1WMQ, 1YTU, 1ZH5, 2A8V, 2DB3, 2G4B, 2HYI, 2J0S, 2JEA, 2JLU, 2PO1, 2Q66, 2VNU, 2XNR, 2XZL, 2XZO, 3D2S, 3ER9, 3EX7, 3FHT, 3G9Y, 3GIB, 3GPQ, 3IEM, 3M7N, 3MDG, 3MJ0, 3NMR, 3O8C, 3PF4, 3QG9, 3QSU, 3SQW, 3T3O, 3T5N, 3U2E, 4AFY, 4G0A, 4H5P, 4HOR, 4I67, 4II9, 4J7L, 4JNG, 4JZU, 4LJ0, 4M59, 4NKU, 4O8J, 4OAV, 4OHY, 4QM6, 4QU6, 4QU7, 4R3I, 4RCJ, 4RCM, 4S2X, 4WAL, 4XWW, 4YOE, 4Z0C, 5A0T, 5AOR, 5BTE, 5BUD, 5C0Y, 5ELH, 5ELK, 5ELT, 5HAB, 5HJZ, 5HK0, 5I9F, 5JJU, 5JRC, 5K77, 5L2L, 5M3H, 5N94, 5O58, 5SZE, 5W0M, 5WWW, 5WWX, 5YKI, 5ZC9, 5ZSA, 5ZUU, 6A4E, 6A6J, 6D1V, 6D2Z, 6E0O, 6E4P, 6EEN, 6FPX, 6FQR, 6GC5, 6I0T, 6IJ2, 6IS0, 6JIM, 6JVX, 6L5N, 6N6A, 6N6I, 6PPQ, 6PUN, 6S0M, 6U6Y, 6UEJ, 6X1B, 6XLV, 6YRB, 6YWO, 6ZM2, 7C45, 7DIC, 7JYY, 7K5L, 7KL3, 7MPL, 7NDJ, 7OM6, 7R9G	1189
tRNA	35	1C0A, 1F7U, 1FFY, 1G59, 1J1U, 1K8W, 1O0B, 1R3E, 1U0B, 2B3J, 2DLC, 2I82, 2RD2, 2ZUE, 3ADD, 3AM1, 3B0U, 3BT7, 3FOZ, 3OVB, 3VJR, 3ZGZ, 4X4T, 4YCO, 4YYE, 4ZDO, 5AH5, 5AXM, 5CCB, 5D0A, 5HR7, 5UD5, 5ZW4, 7K98, 7MJV	490
dsRNA	33	1A34, 1N35, 1YVP, 1YYK, 2GXB, 2ZKO, 3AVX, 3BSN, 3EQT, 3KS8, 3R2D, 3VYX, 3WBM, 4BPB, 4GHA, 4GV3, 4IQX, 4K4X, 4OOG, 4RWN, 4S3N, 4WTJ, 5F8H, 5JAJ, 5W3V, 6LSH, 6M6R, 6O5F, 6SX0, 6TQB, 6VRD, 7KFN, 7OZQ	503
mRNA	24	1GTN, 1KNZ, 2PJP, 2VPL, 2X1A, 2XS2, 3K49, 3NNC, 3PEW, 3Q0Q, 4ED5, 4F02, 4MDX, 4QIK, 4QOZ, 4ZLR, 5BZ1, 5DET, 5M0I, 5UDI, 7A9X, 7CGF, 7NJC, 7S02	264
rRNA	16	1DFU, 1FEU, 1FFK, 1JBR, 1JJ2, 1MJI, 2ASB, 2BH2, 3IEV, 3OIN, 3R9W, 3U4M, 4BW0, 4LGT, 5WZH, 5ZQ8	1047
Aptamer and other folded RNA	16	1OOA, 3AGV, 3AHU, 3DD2, 3EGZ, 3MXH, 4KZD, 4M4O, 4R8I, 5DDP, 5DO4, 5VOE, 6DB8, 6RTI, 6U8D, 7MLX	120
Hairpin	13	1EC6, 1M5K, 2ANN, 2BS0, 2F8K, 2HVY, 2QUX, 2UWM, 4L8H, 6D12, 6DU4, 6F4G, 6FQ3	134
crRNA	13	2XLI, 3QRP, 4AL5, 4C8Y, 4ILL, 5I4A, 5ID6, 5W1H, 5WLH, 6DTD, 6H9H, 6IV8, 7OS0	450
Cyclic RNA	12	1GTF, 6M7K, 6O6V, 6O7B, 6P7P, 6SCE, 6SCF, 6VM6, 6WXQ, 6WXY, 6YUD, 7BDV	168
ncRNA	5	4ATO, 5O1Y, 6DCB, 6HAU, 7K9D	43
Ribozyme	5	1SJ3, 2NZ4, 2R8S, 4PR6, 6XJQ	62
siRNA	5	1RPU, 4NGB, 5JS1, 5THE, 6OON	60
SRP/Srp–like–RNA	5	1DUL, 1JID, 1LNG, 2V3C, 5AOX	62
snRNA/ pre–snRNA	4	4N0T, 4PKD, 5GXH, 6PPN	68
miRNA	4	3ADL, 3TS2, 6MFN, 6UV1	37
piRNA	3	3O7V, 4D25, 5GUH	11
Antitoxin RNA	2	4RMO, 7D8O	209
SsRNA:dsRNA	2	3HJW, 6X5M	13
ctRNA	1	3RW6	16
RNAse–P RNA	1	5XTM	17
Total	329		4963

Table S4. Frequency of different types of proteins present in the 329 crystal structures of RNA–protein complexes.

Protein type	# of structures	PDB codes	Total # of water bridges
Nucleic acid binding protein	115	1C9S, 1EC6, 1FFK, 1GTF, 1GTN, 1RPU, 1SDS, 1YTU, 1YVP, 2ANN, 2F8K, 2G4B, 2HYI, 2XNR, 2XS2, 2ZKO, 3EQT, 3GIB, 3IEV, 3K49, 3MDG, 3MJ0, 3MXH, 3NMR, 3NNC, 3O7V, 3Q0Q, 3QG9, 3QRP, 3TS2, 3WBM, 4BW0, 4C8Y, 4ED5, 4GV3, 4HOR, 4LJ0, 4M59, 4N0T, 4OHY, 4PR6, 4QIK, 4QU6, 4QU7, 4R3I, 4RCJ, 4RCM, 4WAL, 4XWW, 4YOE, 5BZ1, 5DDP, 5DET, 5ELH, 5ELK, 5ELT, 5GXH, 5I4A, 5I9F, 5JAJ, 5JRC, 5L2L, 5SZE, 5THE, 5UDI, 5W1H, 5WLH, 5WWW, 5WWX, 5WZH, 5XTM, 5YKI, 5ZUU, 5ZW4, 6A6J, 6D12, 6E4P, 6EEN, 6FPX, 6FQ3, 6FQR, 6GC5, 6H9H, 6HAU, 6IV8, 6JVX, 6L5N, 6M6R, 6N6A, 6N6I, 6P7P, 6PPN, 6PPQ, 6PUN, 6S0M, 6SCE, 6SCF, 6TQB, 6UV1, 6WXY, 6XLV, 6YUD, 6YWO, 7A9X, 7BDV, 7C45, 7CGF, 7K9D, 7KFN, 7MPL, 7NDJ, 7NJC, 7OS0, 7R9G, 7S02	1587
Hydrolases	73	1B2M, 1JBR, 1YYK, 2B3J, 2DB3, 2GXB, 2J0S, 2JEA, 2JLU, 2PO1, 2VNU, 2XLI, 2XZL, 2XZO, 3DD2, 3EX7, 3FHT, 3IEM, 3M7N, 3O8C, 3PEW, 3R9W, 3SQW, 3T3O, 3VJR, 3VYX, 4AFY, 4AL5, 4BPB, 4D25, 4G0A, 4I67, 4ILL, 4J7L, 4JZU, 4M4O, 4MDX, 4NGB, 4OAV, 4OOG, 4QOZ, 4S2X, 5A0T, 5AOR, 5BTE, 5BUD, 5C0Y, 5DO4, 5GUH, 5HAB, 5HJZ, 5HK0, 5ID6, 5JJU, 5JS1, 5K77, 5N94, 5O58, 5VOE, 6A4E, 6D1V, 6D2Z, 6DTD, 6IJ2, 6MFN, 6O5F, 6OON, 6RTI, 6U6Y, 6VM6, 6VRD, 6ZM2, 7DIC	568
Transferase	32	1N35, 2BH2, 2Q66, 3ADD, 3AM1, 3BSN, 3BT7, 3FOZ, 3OVB, 4II9, 4IQX, 4K4X, 4NKU, 4QM6, 4RWN, 4S3N, 4WTJ, 4X4T, 4ZDO, 5AXM, 5CCB, 5F8H, 5M3H, 5W0M, 5ZQ8, 6DCB, 6DU4, 6E0O, 6I0T, 6IS0, 6LSH, 7MJV	408
Translation / Transcription Protein	22	1M5K, 1OOA, 1SJ3, 1WMQ, 1ZH5, 2A8V, 2ASB, 2PJP, 2UWM, 2VPL, 2X1A, 3AHU, 3AVX, 3ER9, 3G9Y, 3R2D, 4F02, 4ZLR, 5AOX, 5O1Y, 5ZC9, 6WXQ	194
Viral Protein	17	1A34, 1KNZ, 2BS0, 3GPQ, 3KS8, 3T5N, 4GHA, 4H5P, 4JNG, 6JIM, 6SX0, 6X1B, 6YRB, 7JYY, 7K5L, 7KL3, 7OM6	267
Ligase Protein	16	1C0A, 1F7U, 1FFY, 1G59, 1J1U, 1O0B, 1U0B, 2DLC, 2RD2, 2ZUE, 3ZGZ, 4O8J, 4YYE, 5AH5, 5UD5, 7K98	129
Immune system Protein	12	2R8S, 3AGV, 4KZD, 4Z0C, 5ZSA, 6DB8, 6O6V, 6O7B, 6U8D, 6X5M, 6XJQ, 7MLX	216
Ribosomal protein	7	1DFU, 1FEU, 1JJ2, 1MJI, 3OIN, 3U4M, 7OZQ	965
Oxidoreductase Protein	5	3B0U, 4YCO, 5D0A, 5HR7, 6M7K	63
Signaling Protein	4	1DUL, 1JID, 1LNG, 2V3C	39
Lyase Protein	4	1K8W, 1R3E, 2I82, 3U2E	82
Isomerase Protein	3	2HVY, 3HJW, 4LGT	62
Structural Protein	3	2NZ4, 2QUX, 4L8H	15
Toxin Protein	3	4ATO, 4RMO, 7D8O	224
Gene regulation Protein	3	3ADL, 3PF4, 4PKD	23
Anti–Viral Protein	2	5W3V, 6UEJ	28
Transport Protein	2	3RW6, 5M0I	34
Polymerase Protein	1	1UVI	2
snRP	1	3EGZ	7
Metal binding Protein	1	3D2S	11
Chaperone Protein	1	3QSU	0
Splicing Protein	1	6F4G	39
Cytokine Protein	1	4R8I	0
Total	329		4963

Table S5. Distribution of bridging water molecules in terms of the total number of hydrogen bonds formed with amino acid residues and ribonucleotides.

S. No.	# of hydrogen bonds formed by bridging water molecule	# of bridging water molecules
**1.**	2	2372
**2.**	3	1812
**3.**	4	658
**4.**	5*	107
**5.**	6*	13
**6.**	7*	1

*Although a single water molecule cannot theoretically form more than four hydrogen bonds, these possibilities arise due to the involvement of bifurcated hydrogen bonds.

Table S6. Counts of amino acid residues involved in water bridge formation and the associated distribution of hydrogen bonds formed with bridging water in term of main chain and side chain entities.

S. No.	Type of amino acid		Amino acid	# of amino acid residues involved	Total # of hydrogen bonds with bridging water	# of hydrogen bonds of main-chain atoms with bridging water	# of hydrogen bonds of side-chain atoms with bridging water
1.	Nonpolar		Gly	255	287	287	0
2.			Ala	169	184	184	0
3.			Val	135	147	147	0
4.			Leu	158	168	168	0
5.			Ile	91	101	101	0
6.			Met	36	38	31	7
7.			Phe	86	94	94	0
8.			Trp	36	43	32	11
9.			Pro	77	78	78	0
10.	Polar	Uncharged	Ser	442	583	206	377
11.			Asn	481	615	200	415
12.			Cys	51	57	34	23
13.			Gln	312	394	128	266
14.			Thr	333	431	184	247
15.			Tyr	304	340	94	246
16.		Basic	Arg	847	1199	309	890
17.			His	179	209	71	138
18.			Lys	616	789	312	477
19.		Acidic	Asp	442	600	121	479
20.			Glu	288	389	85	304

Table S7. Counts of ribonucleotides involved in water bridge formation and the associated distribution of hydrogen bonds with bridging water in terms of base, sugar, and phosphate moieties.

S. No.	Ribo-nucleotide	# of nucleotides involved	Total # of hydrogen bonds with bridging water	# of hydrogen bonds of base moiety with bridging water	# of hydrogen bonds of ribose moiety with bridging water	# of hydrogen bonds of phosphate moiety with bridging water
**1.**	**rA**	1018	2023	630	717	676
**2.**	**rC**	667	1179	279	385	515
**3.**	**rG**	962	1825	641	552	632
**4.**	**rU**	907	1659	530	530	599

Table S8. Counts of different ribonucleotide atoms involved in water bridge formation.

S. No.	Ribonucleotide moieties		PDB atom nomenclature	Counts
1.	Base	Carbonyl oxygen atoms	*O2*	399
			O4	190
			O6	210
		Endocyclic nitrogen atoms	*N1*	187
			N3	456
			N7	251
		Exocyclic amino nitrogen atoms	*N2*	133
			N4	69
			N6	185
2.	Ribose		*O2'*	1417
			O3'	423
			O4'	253
			O5'	91
3.	Phosphate		*OP1*	1372
			OP2	1050

Table S9. Counts of atoms of different amino acid residues involved in water bridge formation.

S. No.	Amino acid entities	PDB atom nomenclature	Counts
**1.**	**Main chain**	*O*	1810
		N	1048
		OXT	8
**2.**	**Side chain**	*ND1*	39
		ND2	230
		NE	179
		NE1	11
		NE2	225
		NH1	325
		NH2	386
		NZ	477
		OD1	360
		OD2	304
		OE1	274
		OE2	170
		OG	377
		OG1	247
		OH	246
		SD	7
		SG	23

Table S10. Counts of different topologies of water bridges found in the 329 crystal structures.

**S. No.**	**Topology of motifs**	**Sub topologies**	**Counts**		
			**Acyclic**	**Cyclic**	**Total**
**1.**	Triplet	A_1:w:N_1	2374	522	2896
**2.**	Quartet	A_1:w:N_2	481	270	751
		A_2:w:N_1	663	281	944
**3.**	Quintet	A_1:w:N_3	30	18	48
		A_3:w:N_1	69	89	158
		A_2:w:N_2	73	76	149
**4.**	Sextet*	A_1:w:N_4*	1	2	3
		A_2:w:N_3*	0	1	1
		A_3:w:N_2*	0	7	7
		A_4:w:N_1*	2	4	6
**5.**	Total		3693	1270	4963

*Composition-based classification for topologies marked with * is not presented in previous tables, because of their negligible occurrence.

Table S11. Comparison of the counts of ribonucleotide moieties involved in triplet water bridges (A_1:w:N_1) and other higher-ordered topologies.

S. No.	Topology	# of hydrogen bonds of base moiety with bridging water	# of hydrogen bonds of ribose moiety with bridging water	# of hydrogen bonds of phosphate moiety with bridging water
**1.**	Triplet	1088	972	1202
**2.**	Other higher-order topologies	992	1212	1220

Table S12. Comparison of the counts of (main chain or side chain) amino acid moieties involved in triplet water bridge (A_1:w:N_1) and other higher-order topologies.

S. No.	Topology	# of hydrogen bonds of main-chain atoms with bridging water	# of hydrogen bonds of side-chain atoms with bridging water
**1.**	Triplet	1316	1754
**2.**	Other higher order topologies	1550	2126

Table S13. Average B-factors of the different topologies of water bridges.

<table>
<tr><th>S. No.</th><th>Topology</th><th colspan="2">Average B-factor (Å^2)</th></tr>
<tr><td>1.</td><td>A_1:w:N_1</td><td colspan="2">37.3</td></tr>
<tr><td>2.</td><td>A_1:w:N_2</td><td>36.0</td><td rowspan="9">Average for higher-order topologies = 34.9</td></tr>
<tr><td>3.</td><td>A_2:w:N_1</td><td>34.7</td></tr>
<tr><td>4.</td><td>A_1:w:N_3</td><td>34.8</td></tr>
<tr><td>5.</td><td>A_2:w:N_2</td><td>34.5</td></tr>
<tr><td>6.</td><td>A_3:w:N_1</td><td>31.9</td></tr>
<tr><td>7.</td><td>A_1:w:N_4</td><td>25.4</td></tr>
<tr><td>8.</td><td>A_2:w:N_3</td><td>32.9</td></tr>
<tr><td>9.</td><td>A_3:w:N_2</td><td>33.3</td></tr>
<tr><td>10.</td><td>A_4:w:N_1</td><td>28.8</td></tr>
<tr><td colspan="2">Overall average for all topologies</td><td colspan="2">36.3</td></tr>
<tr><td colspan="2">Average for all water molecules</td><td colspan="2">41.4</td></tr>
</table>

Table S14. Frequency distribution of nucleobase-mediated A_1:w:N_1 water bridges, involving the participation of both amino acid chains in water bridge formation, with respect to the identities of the interacting amino acids and their types, and ribonucleotides and their edge types.

		NONPOLAR									POLAR						BASIC			ACIDIC	
		**Gly**	**Ala**	**Val**	**Leu**	**Ile**	**Met**	**Phe**	**Trp**	**Pro**	**Ser**	**Thr**	**Cys**	**Tyr**	**Asn**	**Gln**	**Arg**	**His**	**Lys**	**Asp**	**Glu**
**rA**	**HG**										1				1						
	**WC**																	1		1	1
	**SG**											7								1	1
**rC**	**HG**										2										
	**WC**																			1	
	**SG**										2	1									
**rG**	**HG**														1						
	**WC**										1				1						1
	**SG**										1	1			1						
**rU**	**HG**																				
	**WC**																				
	**SG**														4					1	1

Table S15. Frequency distribution of nucleobase-mediated *cyc*-A_1:w:N_1 water bridges, involving the participation of both amino acid chains in water bridge formation, with respect to interacting and their types, and ribonucleotides and their edge types.

		NONPOLAR									POLAR					BASIC			ACIDIC	
		**Gly**	**Ala**	**Val**	**Leu**	**Ile**	**Met**	**Phe**	**Trp**	**Pro**	**Ser**	**Thr**	**Cys**	**Tyr**	**Asn**	**Gln**	**Arg**	**His**	**Lys**	**Asp**	**Glu**
**rA**	**HG**																				
	**WC**																				
	**SG**										3	1			1					2	1
**rC**	**HG**																				
	**WC**															2					
	**SG**										1										
**rG**	**HG**																				
	**WC**																				
	**SG**																				
**rU**	**HG**																				
	**WC**																				
	**SG**											1									

Table S16. Water bridges using *N7* of rA to interact with the acidic groups of Asp and Glu.

S. No.	PDB	Water	Amino acids	Ribonucleotides
**1.**	1JJ2	6770(0)	OD2-Asp 170(C)	N7-rA 340(0), N6-Ra 340(0)
**2.**	3DD2	1006(B)	OD2-Asp 178(H)	N7-rA 9(B), N7-rG 10(B)
**3.**	4LGT	E2777(E)	OD1-ASP 150(D)	N7-rA 2602(E)
**4.**	4N0T	A0660(A)	OD1-Asp 321(A), NZ-LYS 322(A)	N7-rA 51(B)
**5.**	5W1H	A1679(A)	OD2-Asp 931(A)	N7-rA 6(B)
**6.**	5WLH	A1677(A)	OD2-Asp 931(A)	N7-rA 6(B)
**7.**	6WXQ	B0335(B)	OD1-Asp 121(B)	N7-rA 4(E), N6-rA 4(E)
**8.**	6YUD	J0202(J)	OD2-Asp 69(J)	N7-rA 3(Q), N6-rA 3(Q)
**9.**	6YUD	A0217(A)	OD2-Asp 69(A)	N7-rA 3(K), N6-rA 3(K)
**10.**	6YUD	I0208(I)	OD2-Asp 69(I)	N7-rA 1(Q), N6-rA 1(Q)
**11.**	6YUD	E0216(E)	OD2-Asp 69(E)	N7-rA 3(P), N6-rA 3(P)
**12.**	6YUD	C0206(C)	OD2-Asp 69(C)	N7-rA 1(O), N6-rA 1(O)
**13.**	6YUD	B0208(B)	OD2-Asp 69(B)	N7-rA 1(K), N6-rA 1(K)
**14.**	6YUD	F0211(F)	OD2-Asp 69(F)	N7-rA 1(P), N6-rA 1(P)
**15.**	6YUD	D0214(D)	OD2-Asp 69(D)	N7-rA 3(O), N6-rA 3(O)
**16.**	1JJ2	3838(0)	OE1-Glu 13(J)	N7-rA 1747(0), O2'-rG 1744(0)
**17.**	4PKD	0423(B)	OE2-Glu 244(B)	N7-rA 97(V)
**18.**	5L2L	0632(E)	OE1-Glu 413(E), OD1-Asn 455(E)	N7-rA 3(D)
**19.**	6A6J	0106(D)	OE2-Glu 101(C)	N7-rA 3(D), O2-rC 2(D)
**20.**	7OZQ	0232(E)	OE2-Glu 38(B)	N7-rA 28(E)
**21.**	7OZQ	0217(H)	OE1-Glu 38(A)	N7-rA 28(H)

References:

1. Wild, K., Sinning, I. and Cusack, S. (2001) Crystal structure of an early protein-RNA assembly complex of the signal recognition particle. *Science*, **294**, 598-601.
2. Hainzl, T., Huang, S. and Sauer-Eriksson, A.E. (2007) Interaction of signal-recognition particle 54 GTPase domain and signal-recognition particle RNA in the free signal-recognition particle. *Proc. Natl. Acad. Sci. U. S. A.*, **104**, 14911-14916.
3. Jolma, A., Zhang, J., Mondragón, E., Morgunova, E., Kivioja, T., Laverty, K.U., Yin, Y., Zhu, F., Bourenkov, G., Morris, Q. *et al.* (2020) Binding specificities of human RNA-binding proteins toward structured and linear RNA sequences. *Genome Res.*, **30**, 962-973.
4. Michalska, K., Jedrzejczak, R., Wower, J., Chang, C., Baragaña, B., Gilbert, I.H., Forte, B. and Joachimiak, A. (2021) Mycobacterium tuberculosis Phe-tRNA synthetase: structural insights into tRNA recognition and aminoacylation. *Nucleic Acids Res.*, **49**, 5351-5368.
5. Antson, A.A., Dodson, E.J., Dodson, G., Greaves, R.B., Chen, X.-p. and Gollnick, P. (1999) Structure of the trp RNA-binding attenuation protein, TRAP, bound to RNA. *Nature*, **401**, 235-242.
6. Hoang, C., Chen, J., Vizthum, Caroline A., Kandel, J.M., Hamilton, Christopher S., Mueller, E.G. and Ferré-D'Amaré, A.R. (2006) Crystal Structure of Pseudouridine Synthase RluA: Indirect Sequence Readout through Protein-Induced RNA Structure. *Mol. Cell*, **24**, 535-545.
7. Chao, J.A., Patskovsky, Y., Almo, S.C. and Singer, R.H. (2008) Structural basis for the coevolution of a viral RNA–protein complex. *Nat. Struct. Mol. Biol.*, **15**, 103-105.
8. Kick, L.M., von Wrisberg, M.-K., Runtsch, L.S. and Schneider, S. (2022) Structure and mechanism of the RNA dependent RNase Cas13a from Rhodobacter capsulatus. *Communications Biology*, **5**, 71.
9. Jiao, X., Chang, Jeong H., Kilic, T., Tong, L. and Kiledjian, M. (2013) A Mammalian Pre-mRNA 5′ End Capping Quality Control Mechanism and an Unexpected Link of Capping to Pre-mRNA Processing. *Mol. Cell*, **50**, 104-115.
10. Prabu, J.R., Müller, M., Thomae, Andreas W., Schüssler, S., Bonneau, F., Becker, Peter B. and Conti, E. (2015) Structure of the RNA Helicase MLE Reveals the Molecular Mechanisms for Uridine Specificity and RNA-ATP Coupling. *Mol. Cell*, **60**, 487-499.
11. Fedorov, R., Meshcheryakov, V., Gongadze, G., Fomenkova, N., Nevskaya, N., Selmer, M., Laurberg, M., Kristensen, O., Al-Karadaghi, S., Liljas, A. *et al.* (2001) Structure of ribosomal protein TL5 complexed with RNA provides new insights into the CTC family of stress proteins. *Acta Crystallographica Section D*, **57**, 968-976.
12. Klein, D.J., Schmeing, T.M., Moore, P.B. and Steitz, T.A. (2001) The kink-turn: a new RNA secondary structure motif. *The EMBO Journal*, **20**, 4214-4221.
13. Jiang, Y., Yu, H., Li, F., Cheng, L., Zhu, L., Shi, Y. and Gong, Q. (2018) Unveiling the structural features that determine the dual methyltransferase activities of Streptococcus pneumoniae RlmCD. *PLoS Pathog.*, **14**, e1007379.
14. Zhu, W., McQuarrie, S., Grüschow, S., McMahon, S.A., Graham, S., Gloster, T.M. and White, M.F. (2021) The CRISPR ancillary effector Can2 is a dual-specificity nuclease potentiating type III CRISPR defence. *Nucleic Acids Res.*, **49**, 2777-2789.
15. Krochmal, D., Shao, Y., Li, N.-S., DasGupta, S., Shelke, S.A., Koirala, D. and Piccirilli, J.A. (2022) Structural basis for substrate binding and catalysis by a self-alkylating ribozyme. *Nat. Chem. Biol.*, **18**, 376-384.

16. Teplova, M., Song, J., Gaw, H.Y., Teplov, A. and Patel, D.J. (2010) Structural Insights into RNA Recognition by the Alternate-Splicing Regulator CUG-Binding Protein 1. *Structure*, **18**, 1364-1377.
17. Morgan, C.E., Meagher, J.L., Levengood, J.D., Delproposto, J., Rollins, C., Stuckey, J.A. and Tolbert, B.S. (2015) The First Crystal Structure of the UP1 Domain of hnRNP A1 Bound to RNA Reveals a New Look for an Old RNA Binding Protein. *J. Mol. Biol.*, **427**, 3241-3257.
18. Batey, R.T., Rambo, R.P., Lucast, L., Rha, B. and Doudna, J.A. (2000) Crystal Structure of the Ribonucleoprotein Core of the Signal Recognition Particle. *Science*, **287**, 1232-1239.
19. Özcan, A., Pausch, P., Linden, A., Wulf, A., Schühle, K., Heider, J., Urlaub, H., Heimerl, T., Bange, G. and Randau, L. (2019) Type IV CRISPR RNA processing and effector complex formation in Aromatoleum aromaticum. *Nature Microbiology*, **4**, 89-96.
20. Tunnicliffe, R.B., Levy, C., Ruiz Nivia, H.D., Sandri-Goldin, R.M. and Golovanov, A.P. (2018) Structural identification of conserved RNA binding sites in herpesvirus ORF57 homologs: implications for PAN RNA recognition. *Nucleic Acids Res.*, **47**, 1987-2001.
21. Konno, M., Sumida, T., Uchikawa, E., Mori, Y., Yanagisawa, T., Sekine, S.-i. and Yokoyama, S. (2009) Modeling of tRNA-assisted mechanism of Arg activation based on a structure of Arg-tRNA synthetase, tRNA, and an ATP analog (ANP). *The FEBS Journal*, **276**, 4763-4779.
22. Niewoehner, O., Jinek, M. and Doudna, J.A. (2013) Evolution of CRISPR RNA recognition and processing by Cas6 endonucleases. *Nucleic Acids Res.*, **42**, 1341-1353.
